# Synthesis of Novel FTY720 Analogs with Anticancer Activity through PP2A Activation

**DOI:** 10.3390/molecules23112750

**Published:** 2018-10-24

**Authors:** Jitendra Shrestha, Sung Hwan Ki, Sang Mi Shin, Seon Woong Kim, Joo-Youn Lee, Hee-Sook Jun, Taeho Lee, Sanghee Kim, Dong Jae Baek, Eun-Young Park

**Affiliations:** 1 College of Pharmacy and Natural Medicine Research Institute, Mokpo National University, Jeonnam 58554, Korea; shresthasimon2011@mokpo.ac.kr (J.S.); tjsdnd123@mokpo.ac.kr (S.W.K.); 2 College of Pharmacy, Chosun University, Gwangju, 61452, Korea; shki@chosun.ac.kr (S.H.K.); smshin@chosun.ac.kr (S.M.S.); 3 College of Pharmacy, Seoul National University, Seoul 08826, Korea; leejy@krict.re.kr (J.-Y.L.); pennkim@snu.ac.kr (S.K.); 4 Korea Chemical Bank, Korea Research Institute of Chemical Technology, Daejeon 34114, Korea; 5 Lee Gil Ya Cancer and Diabetes Institute, Department of Molecular Medicine, Gachon University, Incheon 21999, Korea; hsjun@gachon.ac.kr; 6 College of Pharmacy and Gachon Institute of Pharmaceutical Science, Gachon University, Incheon 21936, Korea; 7 College of Pharmacy, Research Institute of Pharmaceutical Sciences, Kyungpook National University, Daegu 41566, Korea; tlee@knu.ac.kr

**Keywords:** FTY720, protein phosphatase 2A, sphingosine kinase, colorectal cancer, derivative

## Abstract

FTY720 inhibits various cancers through PP2A activation. The structure of FTY720 is also used as a basic structure for the design of sphingosine kinase (SK) inhibitors. We have synthesized derivatives using an amide chain in FTY720 with a phenyl backbone, and then compounds were screened by an MTT cell viability assay. The PP2A activity of compound **7** was examined. The phosphorylation levels of AKT and ERK, downstream targets of PP2A, in the presence of compound **7**, were determined. Compound **7** may exhibit anticancer effects through PP2A activation rather than the mechanism by inhibition of SK1 in cancer cells. In the docking study of compound **7** and PP2A, the amide chain of compound **7** showed an interaction with Asn61 that was different from FTY720, which is expected to affect the activity of the compound.

## 1. Introduction

Sphingolipids such as sphingosines, ceramides, glycolipids and sphingosine-1-phosphate (S1P) are important cell signaling lipids that control cellular processes. Sphingolipids play an essential role in cell death and survival, proliferation, recognition and migration [1,2]. S1P binds to a family of five S1P-specific G protein-coupled receptors (GPCRs; named S1P1-5) and regulates the action of various intracellular proteins [3,4]. Sphingosine kinase (SK) has two isoforms: SK1 and 2, and catalyzes sphingosine to S1P. Increased cellular SK and S1P cause cell survival, proliferation and migration [5]. SK inhibitors with various anticancer activities have been developed [6]. However, the relationship between SK2 and cancer is less evident than that of SK1, and it is necessary to develop selective inhibitors for SK1 and SK2. Ceramides modulate protein kinase activities and promote apoptosis through various pathways [7]. Some glycolipids activate natural killer T cells (NKT cells), and they induce various cytokines to suppress cancer or regulate the immune response [8]. Regulation of various sphingolipids is medically available, but there is a continuous requirement for regulators of sphingolipids that are selective for the disease.

**Figure 1:**
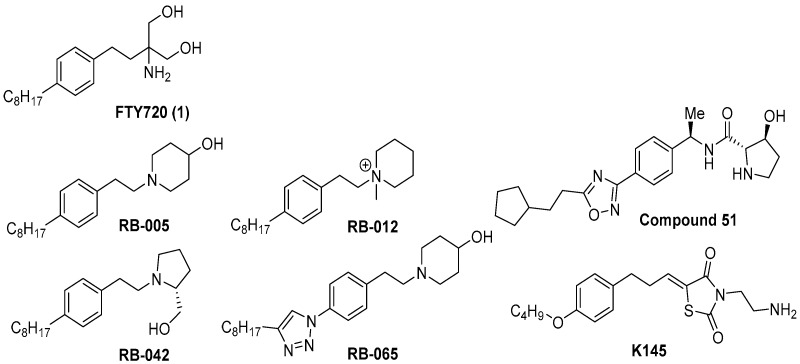
FTY720 (**1**) and known biologically active compounds.

FTY720 (Fingolimod, Gilenya, Novartis) is synthesized based on the structure of myriocine, a fungal metabolite of the Chinese herb *Iscaria sinclarii*. It is structurally and biologically similar to sphingosine. In 2010, FTY720 became the first oral multiple sclerosis treatment approved by the US FDA. FTY720 also activates protein phosphatase 2A (PP2A) and inhibits the growth of various cancer cells (blood cancer, liver cancer, bladder cancer and colorectal cancer) [9,10,11,12]. FTY720 is also known to inhibit SK1 activity by 50% [13]. In its structure, FTY720 has an aromatic backbone, an aliphatic chain and a polar head group. This basic structure of FTY720 has been used as a model on which to design materials for controlling sphingolipids. So far, significant changes in biological activity have been observed in derivatives of the polar head group of FTY720. For example, FTY720 has an inhibitory effect on SK1, but ROME ((*R*)-FTY720 methyl ether) [14], in which the methoxy group is substituted for the hydroxyl group, shows selective inhibition to SK2. However, in the case of ROME, to enantioselectively introduce the methoxy group, 11 synthetic steps are required [15]. In contrast, RB-042 and compound **51** with a pyrrolidine head group are relatively easy to synthesize and yield stereoselective compounds (Figure 1). These compounds exhibit biologically unique activity changes. RB-005 shows selective inhibition of SK1, but RB-042 with (*R*)-(−)-prolinol inhibits both SK1 and SK2 [16,17]. These biological studies provide structural information useful for the development of SK inhibitors. RB-012 with a quaternary ammonium salt inhibited human A549 lung cancer cells, and showed reduced xenograft growth in mice through 14-3-3 protein regulation [18]. Biologically useful results have been observed in the derivatives of the aromatic part of the FTY720 structure. For example, RB-065 is a triazole compound introduced by the click reaction. This compound has the same 4-hydroxypiperidine head structure as RB-005, but it does not inhibit SK2, and it has higher selectivity than RB-005 [17]. Triazole is known as a bioisostere of amide compounds and is widely used in medicinal chemistry [19]. Compound **51** has a structure containing oxadiazole, and it has an IC_50_ of 0.058 μM in SK1 inhibitory activity. The oxadiazole structure exhibited a much higher inhibitory effect than other heterocycle compounds [20]. K145 (3-(2-amino-ethyl)-5-[3-(4-butoxyl-phenyl)-propylidene]-thiazolidine-2,4-dione), which is a backbone structure of phenyl ether, selectively inhibits only SK2 Ki of 6.4 µM. K145 decreases cellular S1P without altering ceramide levels. K145 was found to be orally available and inhibited growth and S1P levels in U937 xenograft tumors in nude mice [21]. The relationship between this structure and activity shows that the backbone structure containing heteroatoms is a useful structure for the design of FTY720 analogues. Although the structure of FTY720 has been studied and applied to various targets, FTY720 derivatives having selectivity to target for pharmaceutical development are required. We synthesized derivatives of FTY720 containing amide chain and investigated whether these derivatives show selectivity to the target.

## 2. Results and Discussion

### 2.1. Chemical Synthesis

**Scheme 1:**
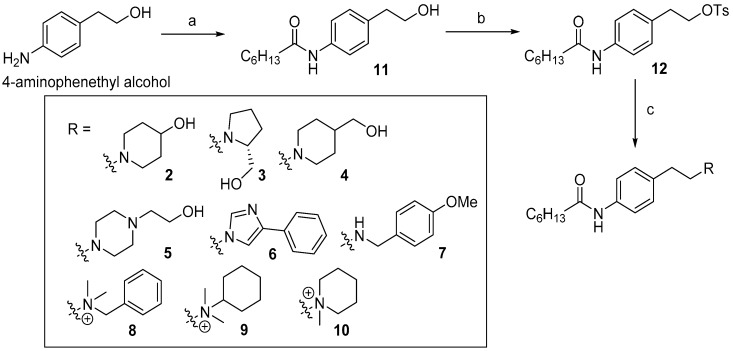
Synthesis of FTY720 derivatives **2**–**10**. Reagents and conditions; (a) heptanoic acid, EDCI, DMAP, CH_2_Cl_2_, rt, 12 h; (b) TsCl, NEt_3_, CH_2_Cl_2_, rt, 6 h; (c) amines, MeCN, 50 °C, 12 h.

4-Aminophenethyl alcohol was used as a starting material to obtain compound **11** in which an acyl chain was introduced by EDCI coupling. Various amines were introduced into the tosylated compound **12** to obtain the final compounds **2**–**10** (Scheme 1).

### 2.2. Compound ***7*** Inhibits the Growth of Human Cancer Cell Lines In Vitro

**Figure 2:**
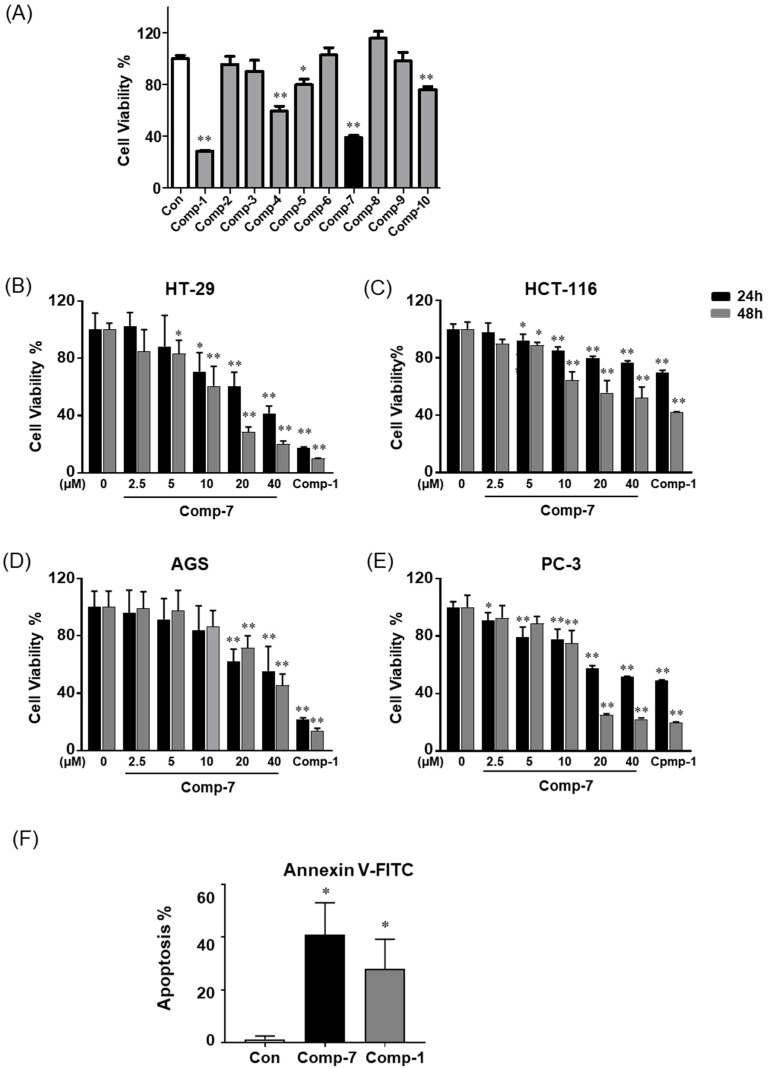
Biological activities of FTY720 derivatives; (**A**) HT-29 cells were treated with compounds **2**–**10** at 40 µM for 24 h (FTY720 (**1**) 20 µM), and cell viability was measured by EZ-CYTOX kit (*n* = 8). Cytotoxic effects of compound **7** and FTY720 in different cancer cells. The 3000 cells/well were plated in a 96-well plate and treated with compound **7** in the concentrations of 2.5, 5, 10, 20 or 40 μM, and FTY720 20 μM in HT-29 cells (**B**), HCT-116 cells (**C**), AGS cells (**D**), and PC-3 cells (**E**); (**F**) annexin V-FITC assays were performed in HT-29 cells. Data are means ± SD from three independent experiments. * *p* < 0.05, ** *p* < 0.01 compared with control cells.

We performed an MTT assay on human colorectal cancer line HT-29 in order to investigate the cell cytotoxic effects of newly synthesized compounds **2**–**10**. We found that the greatest cell death effects were observed in compound **7** (Figure 2A). In order to observe the cytotoxic effect of compound **7** in various cancer cell lines, we investigated the cell viability assay in human colorectal cancer lines HT-29 and HCT-116, human gastric cancer cell line AGS and human prostate cancer cell line PC-3 in the concentration range of 2.5, 5, 10, 20 and 40 μM along with 20 μM FTY720. The result of the MTT assays shows that compound **7** significantly inhibits the growth of all human cancer cells in vitro in a dose- and time-dependent manner (Figure 2B–E). We estimated the IC_50_ of compound **7** in cancer cell lines (Table 1). To examine whether the cytotoxic effects in the compound **7**-treated cell might be a result of the increased apoptosis, we performed an annexin V-FITC cell apoptosis assay. We found that incubation of HT-29 cells with compound **7** for 24 h resulted in a significant increase in the number of apoptotic cells relative to the number of untreated control cells (Figure 2F).

### 2.3. Effect of PF-543 and Compound ***7*** on SK1 Activity

In order to investigate whether the cytotoxic effect of compound **7** mediates the inhibition of SK1 activity, the SK1 inhibitory effect was tested. As a result, compound **7** inhibited SK1 activity by about 15% compared with control, whereas the conventional SK1 inhibitor PF-543 [22] inhibited SK1 activity by about 45%. These results suggest that compound **7** induces antitumor activity through a mechanism other than SK1 activity inhibition (Supplementary Data Figure S1).

### 2.4. Compoud ***7*** Effectively Activates PP2A and Decreases the Phosphorylation of ERK and AKT

**Figure 3:**
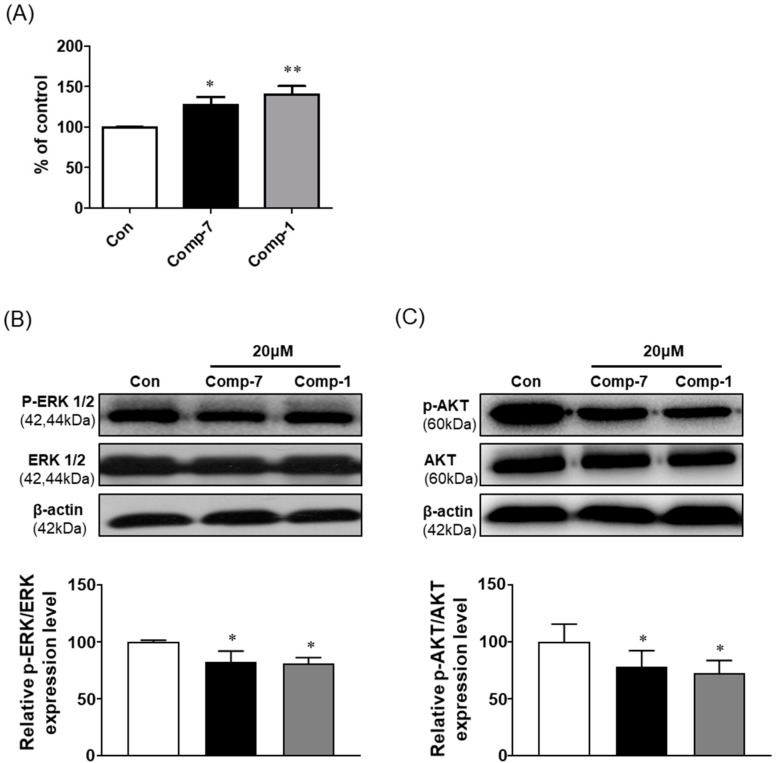
PP2A activity of compound **7** in HT-29 cells. (**A**) PP2A activity was measured in HT-29 cells treated with the 20 μM of compound **7** and compound **1** (FTY720) for 12 h. (**B**,**C**) Cells were treated with or without 20 μM compound **7** and FTY720 for 12 h, protein extracted, separated, and probed with pERK1/2, ERK1/2, pAKT and AKT. The band was analyzed by UVITEC imaging software, and data were plotted as means ± SD for three replicates of the experiments. * *p* < 0.05, ** *p* < 0.01 compared with control cells.

Protein phosphatase 2A (PP2A) is a main protein serine/threonine phosphatase, maintains the signaling homeostasis of cells through dephosphorylation of several signaling proteins, and inhibits tumor growth [23]. In colorectal cancer, the activity of PP2A is inhibited, and FTY720 effectively activates the functions of PP2A [24]. In this study, we performed the PP2A activity assay in HT-29 cells treated with compound **7**. The PP2A activity was more significantly increased in compound **7**-treated cell lysate than the untreated control groups (Figure 3A). Furthermore, we performed the Western blotting of phosphorylation of ERK and AKT, which are known to be dephosphorylated by PP2A. Compared to the control, the phosphorylation of ERK and AKT was significantly decreased in the compound **7**-treated group without affecting the total expression level (Figure 3B,C). These results suggest that compound **7** may exhibit anticancer effect through PP2A activation in cancer cells.

### 2.5. Molecular Modeling Studies of FTY720 and Compound ***7*** Against the Human I2PP2A/SET

**Figure 4:**
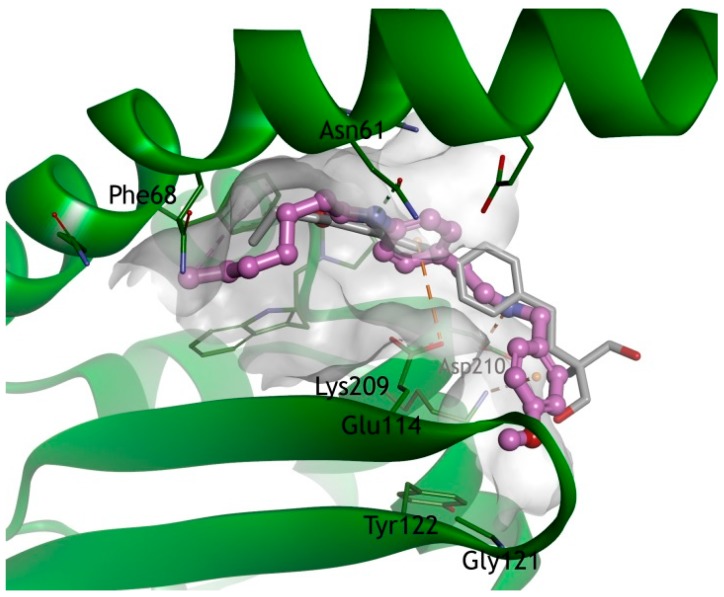
Docking model of FTY720 (**1**, gray, ball and stick) and compound **7** (purple, ball and stick) in the I2PP2A/SET. The X-ray structure of IPP2A/SET was obtained from the Protein Data Bank (PDB code 2E50). The I2PP2A/SET is represented by a blue ribbon model. The hydrogen bonds are shown as a green dashed line, and an orange dashed line represents electrostatic interactions. Also, the hydrophobic interactions are shown as a pink dashed line.

A docking study was conducted to determine if compound **7** effectively binds to PP2A (PDB code 2E50) [25]. As the hydroxy group of FTY720 interacts with Glu206 and Lys209, the methoxyphenyl group, which is the head group of compound **7**, interacts with the Lys209 (cation-pi interaction). The secondary amine group of compound **7** interacts with Asp210, and the amide linker interacts with Asn61. The terminal acyl chain of compound **7** showed interactions with Phe68, as FTY720 (Figure 4). 

## 3. Experimental Section

### 3.1. Synthesis in General

All chemicals were reagent grade and used as purchased. Reactions were run under nitrogen and were monitored by TLC using silica gel 60 F254 aluminum-backed plates. Flash column chromatography was performed on silica gel grade 60 (230−400 mesh). All solvents were of anhydrous quality and were used as received. A Bruker Avance I spectrometer was used to record ^1^H NMR and ^13^C NMR spectra, and chemical shifts were reported in δ units relative to deuterated solvents, which served as internal references, at 400 and 100 MHz, respectively. High-resolution mass spectra were recorded on an Agilent Technologies G6520A Q-TOF mass spectrometer using electrospray ionization (ESI). By examining their HRMS and ^1^H NMR spectra, all compounds were determined to be ≥95% pure.

### 3.2. Synthesis

*N-(4-(2-Hydroxyethyl)phenyl)heptanamide* (**11**) EDCI (1.7 g, 0.0087 mol), DMAP (45 mg, 0.36 mmol), and heptanoic acid (0.95 g, 0.0073 mol) were added to a stirred solution of 4-aminophenethyl alcohol (1.0 g, 0.0073 mol) in CH_2_Cl_2_ (15 mL). The reaction mixture was stirred under nitrogen for 12 h. The solvent was removed in vacuo and the resulting residue was purified by flash column chromatography to afford the desired products **11** (1.6 g, 90%). ^1^H NMR (400 MHz, CDCl_3_) δ 7.30 (d, *J* = 8.3 Hz, 2H), 6.99 (d, *J* = 8.3 Hz, 2H), 3.61 (t, *J* = 7.0 Hz, 2H), 2.65 (t, *J* = 7.0 Hz, 2H), 2.19 (t, *J* = 7.6 Hz, 2H), 1.61–1.45 (m, 2H), 1.26–1.09 (m, 6H), 0.74 (t, *J* = 6.4 Hz, 3H); ^13^C NMR (100 MHz, CDCl_3_) δ 173.4, 136.8, 135.0, 129.6(2C), 120.8(2C), 63.6, 38.9, 37.6, 31.9, 29.3, 26.1, 22.9, 14.3; ESI-HRMS (M + H)^+^ *m*/*z* calcd. for C_15_H_24_NO_2_ 250.1807, found 250.1844.

*4-Heptanamidophenethyl 4-methylbenzenesulfonate* (**12**) To a solution of **11** (1.35 g, 0.0054 mol) and triethylamine (3.77 mL, 0.027 mol) in CH_2_Cl_2_ (30 mL) at 0 °C was added p-toluenesulfonyl chloride (1.54 g, 0.0082 mol). After being stirred at room temperature for 6 h, the reaction mixture was evaporated, diluted with water, and the product was extracted with EtOAc. The extract was washed with brine, dried, and evaporated. Flash column chromatography with hexane/EtOAc (10:1) as the eluent gave compound **12** (2.0 g, 94%). ^1^H NMR (400 MHz, CDCl_3_) δ 7.65 (d, *J* = 8.3 Hz, 2H), 7.39 (d, *J* = 8.4 Hz, 2H), 7.26 (d, *J* = 8.0 Hz, 2H), 7.00 (d, *J* = 8.4 Hz, 2H), 4.14 (t, *J* = 7.0 Hz, 2H), 2.87 (t, *J* = 7.0 Hz, 2H), 2.40 (s, 3H), 2.35–2.29 (m, 2H), 1.72–1.62 (m, 2H), 1.38–1.22 (m, 6H), 0.85 (t, *J* = 6.9 Hz, 3H); ^13^C NMR (100 MHz, CDCl_3_) δ 172.2, 145.2, 137.3, 133.2, 132.4, 130.3(2C), 129.8(2C), 128.2(2C), 120.5(2C), 71.1, 38.1, 35.1, 32.0, 29.3, 26.1, 22.9, 22.0, 14.5; ESI-HRMS (M + H)^+^ *m*/*z* calcd. for C_22_H_30_NO_4_S 404.1896, found 404.1858.

*N-(4-(2-(4-Hydroxypiperidin-1-yl)ethyl)phenyl)heptanamide, 4-methylbenzenesulfonate salt* (**2**) To a solution of **12** (50 mg, 0.12 mmol) in 7 mL of acetonitrile was added 4-hydroxypiperidine (38 mg, 0.37 mmol). The reaction mixture was stirred at 50 °C for 12 h and concentrated. Purification by silica gel chromatography, eluting with CH_2_Cl_2_/MeOH (10:1), gave 44 mg (73%) of compound **2** as a slightly yellow waxy solid. ^1^H NMR (400 MHz, MeOD/CDCl_3_ = 3/1) δ 7.52 (d, *J* = 8.2 Hz, 2H), 7.28 (d, *J* = 8.5 Hz, 2H), 6.99 (d, *J* = 8.0 Hz, 2H), 6.91 (d, *J* = 8.5 Hz, 2H), 3.57 (s, OH), 2.89 (t, *J* = 7.8 Hz, 2H), 2.70–2.63 (m, 5H), 2.53–2.47 (m, 2H), 2.15 (s, 3H), 2.13 (t, *J* = 7.6 Hz, 2H), 1.86–1.73 (m, 2H), 1.60–1.41 (m, 4H), 1.19–1.01 (m, 6H), 0.67 (t, *J* = 6.8 Hz, 3H); ^13^C NMR (100 MHz, MeOD/CDCl_3_ = 3/1) δ 175.0, 143.1, 142.6, 138.9, 130.7(4C), 127.5(2C), 122.2(2C), 61.0, 51.3, 38.9, 33.7, 33.3, 33.0, 30.7, 27.5, 24.2, 22.9, 15.6; ESI-HRMS (M+H)^+^ *m*/*z* calcd. for C_20_H_33_N_2_O_2_ 333.2542, found 333.2575.

*(R)-N-(4-(2-(2-(Hydroxymethyl)pyrrolidin-1-yl)ethyl)phenyl)heptanamide, 4-methylbenzenesulfonate salt* (**3**) Compound **3** was prepared from **12** and (R)-(−)-prolinol according to the same reaction procedure as that described for **1**. Yield = 76%; ^1^H NMR (400 MHz, MeOD/CDCl_3_ = 3/1) δ 7.54 (d, *J* = 8.2 Hz, 2H), 7.30 (d, *J* = 8.6 Hz, 2H), 7.00 (d, *J* = 8.5 Hz, 2H), 6.95 (d, *J* = 8.5 Hz, 2H), 3.63 (qd, *J* = 12.5, 5.3 Hz, 2H), 3.54–3.46 (m, 1H), 3.39 (td, *J* = 11.9, 5.5 Hz, 1H), 3.31 (dd, *J* = 10.5, 4.2 Hz, 1H), 2.97 (td, *J* = 11.3, 5.6 Hz, 1H), 2.82 (dddd, *J* = 20.8, 18.8, 13.2, 5.4 Hz, 3H), 2.16 (s, 3H), 2.15 (t, *J* = 8.0 Hz, 2H), 2.03–1.91 (m, 1H), 1.85 (dd, *J* = 13.7, 7.5 Hz, 2H), 1.67 (td, *J* = 13.5, 6.8 Hz, 1H), 1.49 (dt, *J* = 15.3, 7.6 Hz, 2H), 1.22–1.04 (m, 6H), 0.68 (t, *J* = 6.7 Hz, 3H); ^13^C NMR (100 MHz, MeOD/CDCl_3_ = 3/1) δ 173.6, 141.8, 141.0, 137.9, 132.0, 129.3(4C), 126.1(2C), 120.9(2C), 60.8, 55.1, 37.5, 31.9, 31.8, 29.3, 26.6, 26.1, 23.2, 22.8, 21.4, 14.2; ESI-HRMS (M+H)^+^ *m*/*z* calcd. for C_20_H_33_N_2_O_2_ 333.2542, found 333.2517.

*N-(4-(2-(4-(Hydroxymethyl)piperidin-1-yl)ethyl)phenyl)heptanamide, 4-methylbenzenesulfonate salt* (**4**) Compound **4** was prepared from **12** and 4-piperidinemethanol according to the same reaction procedure as that described for **1**. Yield = 82%; ^1^H NMR (400 MHz, MeOD/CDCl_3_ = 3/1) δ 7.61 (d, *J* = 8.2 Hz, 2H), 7.35 (d, *J* = 8.4 Hz, 2H), 7.07 (d, *J* = 8.1 Hz, 2H), 6.98 (d, *J* = 8.4 Hz, 2H), 3.30 (d, *J* = 6.0 Hz, 4H), 2.94–2.87 (m, 2H), 2.87–2.78 (m, 2H), 2.52 (t, *J* = 11.1 Hz, 2H), 2.23 (s, 3H), 2.22–2.17 (m, 2H), 1.76 (d, *J* = 12.6 Hz, 2H), 1.60–1.37 (m, 5H), 1.25–1.10 (m, 6H), 0.75 (t, *J* = 6.7 Hz, 3H); ^13^C NMR (100 MHz, MeOD/CDCl_3_ = 3/1) δ 174.8, 143.2, 142.5, 139.1, 130.8(4C), 127.5(2C), 122.2(2C), 67.4, 54.7, 39.0, 38.3, 33.3, 32.2, 30.7, 27.8, 24.3, 23.0, 15.7; ESI-HRMS (M + H)^+^ *m*/*z* calcd. for C_21_H_35_N_2_O_2_ 347.2699, found 347.2629.

*N-(4-(2-(4-(2-Hydroxyethyl)piperazin-1-yl)ethyl)phenyl)heptanamide, 4-methylbenzenesulfonate salt* (**5**) Compound **5** was prepared from **12** and 1-(2-hydroxyethyl)piperazine according to the same reaction procedure as that described for **1**. Yield = 62%; ^1^H NMR (400 MHz, MeOD/CDCl_3_ = 3/1) δ 7.59 (d, *J* = 8.2 Hz, 2H), 7.35 (d, *J* = 8.4 Hz, 2H), 7.08 (d, *J* = 8.0 Hz, 2H), 6.99 (d, *J* = 8.4 Hz, 2H), 3.52 (t, *J* = 5.6 Hz, 2H), 2.63 (dd, *J* = 10.3, 6.1 Hz, 2H), 2.56–2.38 (m, 12H), 2.23 (s, 3H), 2.22–2.17 (m, 2H), 1.59–1.49 (m, 2H), 1.26–1.12 (m, 6H), 0.75 (t, *J* = 6.8 Hz, 3H); ^13^C NMR (100 MHz, MeOD/CDCl_3_ = 3/1) δ 174.9, 143.4, 142.5, 138.5, 137.1, 130.7(4C), 127.5(2C), 122.1(2C), 60.0, 38.9, 34.3, 33.4, 30.7, 27.5, 24.3, 22.9, 15.7; ESI-HRMS (M + H)^+^ *m*/*z* calcd. for C_21_H_36_N_3_O_2_ 362.2808, found 362.2833.

*N-(4-(2-(4-Phenyl-1H-imidazol-1-yl)ethyl)phenyl)heptanamide, 4-methylbenzenesulfonate salt* (**6**) Compound **6** was prepared from **12** and 4-phenylimidazole according to the same reaction procedure as that described for **1**. Yield = 54%; ^1^H NMR (400 MHz, MeOD/CDCl_3_ = 3/1) δ 8.69 (s, NH), 7.49 (d, *J* = 8.1 Hz, 2H), 7.29 (d, *J* = 5.6 Hz, 1H), 7.27 (d, *J* = 4.4 Hz, 1H), 7.23 (d, *J* = 7.5 Hz, 2H), 7.16 (d, *J* = 8.2 Hz, 1H), 6.95 (d, *J* = 8.0 Hz, 2H), 6.88 (d, *J* = 7.3 Hz, 2H), 6.83–6.79 (m, 1H), 6.76 (d, *J* = 8.2 Hz, 2H), 6.46 (d, *J* = 8.2 Hz, 1H), 4.18 (t, *J* = 5.9 Hz, 1H), 4.09 (t, *J* = 5.4 Hz, 1H), 2.83 (t, *J* = 5.5 Hz, 1H), 2.55 (t, *J* = 6.0 Hz, 1H), 2.15–2.07 (m, 2H), 2.11 (s, 3H), 1.51–1.36 (m, 2H), 1.16–0.97 (m, 6H), 0.63 (t, *J* = 6.2 Hz, 3H); ^13^C NMR (100 MHz, MeOD/CDCl_3_ = 3/1) δ 175.2, 143.2, 142.4, 139.6, 138.2, 137.2, 133.0, 132.6, 132.5, 131.2, 131.1, 130.8, 130.6, 127.5, 126.4, 122.4, 122.3, 121.6, 52.8, 50.4, 38.9, 37.3, 37.0, 33.3, 30.7, 27.5, 24.2, 22.8; ESI-HRMS (M + H)^+^ *m*/*z* calcd. for C_24_H_30_N_3_O 376.2389, found 376.2391.

*N-(4-(2-((4-Methoxybenzyl)amino)ethyl)phenyl)heptanamide* (**7**) Compound **7** was prepared from **12** and 4-methoxybenzylamine according to the same reaction procedure as that described for **1**. Yield = 72%; ^1^H NMR (400 MHz, MeOD/CDCl_3_ = 3/1) δ 7.39 (d, *J* = 8.4 Hz, 2H), 7.10 (d, *J* = 8.6 Hz, 2H), 7.03 (d, *J* = 8.4 Hz, 2H), 6.78 (d, *J* = 8.6 Hz, 2H), 3.71 (s, 3H), 3.64 (s, 2H), 2.81–2.73 (m, 2H), 2.70 (dd, *J* = 10.9, 4.3 Hz, 2H), 2.26 (t, *J* = 7.6 Hz, 2H), 1.66–1.55 (m, 2H), 1.34–1.18 (m, 6H), 0.80 (t, *J* = 6.8 Hz, 3H); ^13^C NMR (100 MHz, MeOD/CDCl_3_ = 3/1) δ 174.9, 160.7, 138.6, 136.5, 132.2, 131.5(2C), 130.7(2C), 122.1(2C), 115.7(2C), 56.9, 54.4, 51.5, 38.9, 36.5, 33.3, 30.7, 27.5, 24.2, 15.6; ESI-HRMS (M + H)^+^ *m*/*z* calcd. for C_23_H_33_N_2_O_2_ 369.2542, found 369.2538.

*N-Benzyl-2-(4-heptanamidophenyl)-N,N-dimethylethan-1-aminium 4-methylbenzenesulfonate* (**8**) Compound **8** was prepared from **12** and *N*-benzyldimethylamine according to the same reaction procedure as that described for **1**. Yield = 66%; ^1^H NMR (400 MHz, MeOD/CDCl_3_ = 3/1) δ 7.71 (d, *J* = 8.2 Hz, 2H), 7.52 (d, *J* = 8.5 Hz, 2H), 7.49–7.40 (m, 5H), 7.15 (t, *J* = 8.2 Hz, 4H), 4.54 (s, 2H), 3.50–3.41 (m, 2H), 3.11–3.01 (m, 2H), 3.08 (s, 6H), 2.37–2.24 (m, 2H), 2.32 (s, 3H), 1.72–1.60 (m, 2H), 1.39–1.21 (m, 6H), 0.87 (t, *J* = 6.8 Hz, 3H); ^13^C NMR (100 MHz, MeOD/CDCl_3_ = 3/1) δ 175.0, 146.9, 144.1, 142.0, 139.7, 139.1, 134.6, 134.2, 133.5, 132.7, 131.7, 131.1, 131.0, 130.5, 129.5, 128.7, 127.4, 122.4, 122.0, 72.8, 70.0, 66.7, 38.9, 36.4, 33.3, 30.6, 30.2, 27.4, 24.2, 22.8, 15.6; ESI-HRMS (M + H)^+^ *m*/*z* calcd. for C_24_H_36_N_2_O 368.2822, found 368.2809.

*N-(4-Heptanamidophenethyl)-N,N-dimethylcyclohexanaminium 4-methylbenzenesulfonate* (**9**) Compound **9** was prepared from **12** and *N*,*N*-dimethylcyclohexylamine according to the same reaction procedure as that described for **1**. Yield = 63%; ^1^H NMR (400 MHz, MeOD/CDCl_3_ = 3/1) δ 7.49 (d, *J* = 8.2 Hz, 2H), 7.31 (d, *J* = 8.5 Hz, 2H), 6.95 (dd, *J* = 8.1, 5.4 Hz, 4H), 3.93 (t, *J* = 6.8 Hz, 1H), 3.27–3.19 (m, 2H), 2.84 (s, 6H), 2.81–2.73 (m, 2H), 2.65 (t, *J* = 6.8 Hz, 1H), 2.11 (s, 3H), 2.13–2.09 (m, 3H), 1.94 (d, *J* = 10.5 Hz, 2H), 1.77 (d, *J* = 13.4 Hz, 2H), 1.54–1.40 (m, 4H), 1.29–1.05 (m, 8H), 0.65 (t, *J* = 6.8 Hz, 3H); ^13^C NMR (100 MHz, MeOD/CDCl_3_ = 3/1) δ 174.9, 146.8, 144.0, 141.8, 139.7, 130.4(4C), 127.4(2C), 122.4(2C), 74.5, 65.1, 38.8, 33.2, 30.6, 29.9, 27.8, 27.4, 27.0, 26.3, 24.1, 23.0, 22.7, 15.5; ESI-HRMS (M + H)^+^ *m*/*z* calcd. for C_23_H_40_N_2_O 360.3135, found 360.3123.

*1-(4-Heptanamidophenethyl)-1-methylpiperidin-1-ium 4-methylbenzenesulfonate* (**10**) Compound **10** was prepared from **12** and 1-methylpiperidine according to the same reaction procedure as that described for **1**. Yield = 90%; ^1^H NMR (400 MHz, MeOD/CDCl_3_ = 3/1) δ 7.54 (d, *J* = 8.1 Hz, 2H), 7.36 (d, *J* = 8.4 Hz, 2H), 7.00 (d, *J* = 6.6 Hz, 4H), 3.98 (t, *J* = 6.8 Hz, 1H), 3.37–3.25 (m, 1H), 3.21 (t, *J* = 5.7 Hz, 2H), 2.93 (s, 3H), 2.81 (dd, *J* = 10.1, 6.7 Hz, 1H), 2.70 (t, *J* = 6.8 Hz, 1H), 2.19–2.13 (m, 2H), 2.17 (s, 3H), 1.67 (dd, *J* = 10.7, 5.6 Hz, 4H), 1.59–1.42 (m, 4H), 1.24–1.06 (m, 8H), 0.70 (t, *J* = 6.8 Hz, 3H); ^13^C NMR (100 MHz, MeOD/CDCl_3_ = 3/1) δ 175.0, 146.9, 142.0, 139.7, 139.1, 130.6(2C), 129.5(2C), 127.5(2C), 122.4(2C), 63.0, 38.9, 36.4, 33.3, 30.6, 29.4, 27.4(2C), 24.2, 23.2, 22.8, 22.5, 21.7, 15.6; ESI-HRMS (M + H)^+^ *m*/*z* calcd. for C_21_H_36_N_2_O 332.2822, found 332.2871.

### 3.3. Chemicals, Reagents and Antibodies

DMEM, RPMI, fetal bovine serum (FBS), penicillin–streptomycin and trypsin 0.25% were purchased from GE Healthcare Life Sciences HyClone Laboratories (Pittsburgh, PA, USA). EZ-CYTOX was obtained from DoGenBio Co. Ltd. (Seoul, Korea). ApoScan^TM^ annexin V-FITC apoptosis detection kit was received from Biobud (Cat. No.: LS-02-100, Gyeonggi-do, Korea). PP2A activity assay kit was acquired from Millipore Corporation (Billerica, MA, USA). SK1 assay kit was received from Echelon Corporation (Santa Clara, CA, USA). AKT antibody was purchased from Cell Signaling Technology (Danvers, MA, USA). Anti-ERK, anti-pERK, anti-pAKT, anti-β-actin, and anti-HRP conjugated anti-rabbit and anti-mouse secondary antibodies were obtained from Santa Cruz Biotechnology (Dallas, TX, USA). Protein marker was received from Thermo Fisher Scientific (Waltham, MA, USA) Western blotting ECL solution was obtained from Millipore Corporation (Burlington, MA, USA)

### 3.4. Cancer Cell Line and Culture

Colorectal cancer cell lines (HT-29 and HCT-116), gastric cancer cell line (AGS) and prostate cancer cell line (PC-3) were purchased from Korean Cell Line Bank. Cells were cultured in RPMI/DMEM medium supplied with 10% FBS, streptomycin (100 U/mL) and penicillin (100 U/mL) in 95% humidified, 5% CO_2_ incubator at 37 °C.

### 3.5. MTT Cell Viability Assay

Cancer cell viability assay was performed by 3-[4,5-dimethylthiazol-2-yl]-2,5 diphenyltetrazolium bromide (MTT) assay. Briefly, subsequent 3000 cells/well were plated in 96-well plates, after 24/48 h treatment of derivatives and FTY720, EZ-CYTOX 10 μL was added and incubated for 90 min. Absorbance was recorded using Thermo Scientific Multiskan GO (Waltham, MA, USA) at 450 nm wavelengths. 

### 3.6. Annexin V Assay

Cell apoptosis assay was determined using annexin V-FITC and propidium iodide (PI) staining as described in the manufacturer’s instructions. Briefly, HT-29 cells (5 × 10^5^/well) were seeded in 12-well plates. Cells were treated with compound **7** for 24 h and cell apoptosis was analyzed by annexin V-FITC staining. Data analysis was performed using an Arthur image-based cytometer. 

### 3.7. PP2A Activity Assay

PP2A activity assay was a PP2A immunoprecipitation phosphatase assay kit operated according to the manufacturer’s instruction. Briefly, prepared protein aliquot was incubated with anti-PP2A subunit C-antibody and protein A agarose slurry at 4 °C for 2–3 h, and then phosphopeptide was added. Samples were recorded by Thermo Scientific Multiskan GO. 

### 3.8. Immunoblotting

Cell extracts from HT-29 cells treated with compound **7** and FTY720 were prepared. After protein quantification, protein samples (20–30 μg/lane) were separated by electrophoresis and then transferred to Immobilon-P PVDF membranes. The membranes were incubated with anti-β-actin, anti-ERK, anti-pERK, anti-AKT and anti-pAKT antibodies. The primary antibodies were detected using horseradish peroxidase-conjugated anti-mouse IgG with UVITEC or X-ray film. 

### 3.9. Molecular Modeling Studies

Molecular modeling studies of FTY720 and compound **7** against the human I2PP2A/SET were performed using Schrödinger Suite 2017-2 (Schrödinger, LLC, http://www.schrodinger.com). The X-ray crystal structure was obtained from the Protein Data Bank (http://www.rcsb.org/pdb, PDB code: 2E50). The protein preparation was revised using Protein Preparation Wizard in Maestro v.11.2 and the receptor grid box was generated as 25 × 25 × 25 Å cubic size centered on complexed ligand. The ligands were minimized using OPLS_2005 force field with a dielectric constant value 80.0 in MacreoModel v.11.6. Flexible dockings were performed using the Glide v.7.5 program with standard precision method. The docking models of ligands were visualized using Discovery Studio 2016 (http://www.biovia.com). 

### 3.10. Statistical Analysis

The results were analyzed using GraphPad Prism 7 with three independent experiments (mean ± SD). One-way ANOVA with post-hoc analysis using the Newman-Keuls multiple comparison test was used to analyze parametric data. Results were accepted as significant for *p*-values < 0.05. 

## 4. Conclusions

We have synthesized new materials with very few synthetic steps, unlike the synthesis of FTY720 and its derivatives. Among the newly synthesized compounds, compound **7** is considered to have the most effective anticancer property, and the activity of this property was thought to be due to PP2A activation rather than the mechanism leading to inhibition of SK1. Docking studies in PP2A of FTY720 and compound **7** showed that the aromatic head group of compound **7** could effectively replace the complex structure of the head group of FTY720. In addition, the amide linker of compound **7** is expected to show good activity results by interacting with Asn61, which does not interact with FTY720. These biological results and the structure of the compounds provide important information for the design of new sphingolipid-based compounds targeting PP2A.

## Figures and Tables

**Table 1:** Cytotoxic activity of compound **7**. IC_50_ values for each cell line were calculated by using GraphPad Prism 7.

Compound	IC_50_ (µM) 24 h			
	HT-29	HCT-116	AGS	PC-3
Compound **7**	10.39	7.335	11.04	7.456

## Supplementary Materials

The following are available online: Effect of PF-543 and compound **7** on SK1 activity, cell cytotoxicity of compound **7** in cancer and normal cell line and copies of ^1^H NMR, ^13^C NMR and HRMS spectra for compounds.
