# Supplementary material for: Synthesis of Novel FTY720 Analogs with Anticancer Activity through PP2A Activation
Source: Molecules. 2018 Oct 24;23(11):2750. doi: 10.3390/molecules23112750 (PMC6278267; doi:10.3390/molecules23112750)

Supplementary data

Synthesis of Novel FTY720 Analogs with Anticancer Activity through PP2A Activation

Jitendra Shrestha [1†], Sung Hwan Ki [2†], Sang Mi Shin [2], Seon Woong Kim [1], Joo-Youn Lee [3,4], Hee-Sook Jun [5], Taeho Lee [6], Sanghee Kim [3], Dong Jae Baek [1,*], Eun-Young Park [1,*]

1 College of Pharmacy and Natural Medicine Research Institute, Mokpo National University, Jeonnam 58554, South Korea; shresthasimon2011@mokpo.ac.kr (J.S.); tjsdnd123@mokpo.ac.kr (S.W.K.)
2 College of Pharmacy, Chosun University, Gwangju, 61452, South Korea; shki@chosun.ac.kr (S.H.K.); smshin@chosun.ac.kr (S.M.S.)
3 College of Pharmacy, Seoul National University, Seoul 08826, South Korea; leejy@krict.re.kr (J.-Y.L.); pennkim@snu.ac.kr (S.K.)
4 Korea Chemical Bank, Korea Research Institute of Chemical Technology, Daejeon 34114, South Korea; leejy@krict.re.kr (J.-Y.L.)
5 Lee Gil Ya Cancer and Diabetes Institute, Department of Molecular Medicine, Gachon University, Incheon 21999, South Korea; College of Pharmacy and Gachon Institute of Pharmaceutical Science, Gachon University, Incheon 21936, South Korea; hsjun@gachon.ac.kr (H.-S.J.)
6 College of Pharmacy, Research Institute of Pharmaceutical Sciences, Kyungpook National University, Daegu 41566, South Korea; tlee@knu.ac.kr (T.L.)

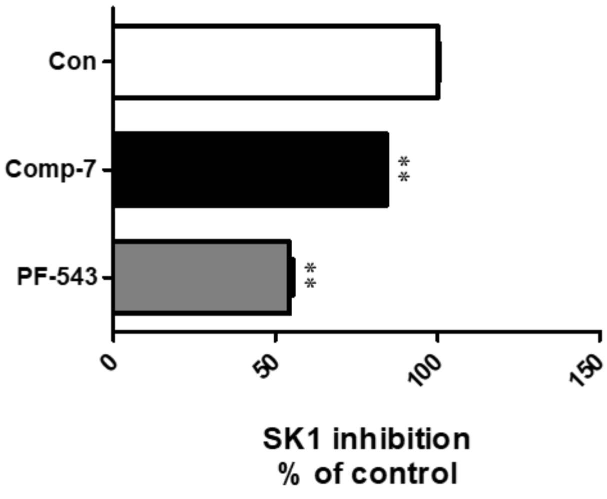

Figure S1. SK1 inhibition assay of compound **7.** SK1 activity was measured with 40 μM compound **7** (Comp-7) and PF-543 using Echelon's Sphingosine Kinase Activity Assay kit according to the manufacturer's protocol (100 μM sphingosine, 10 μM ATP and 0.5 ng/μL of recombinant sphingosine kinase 1).

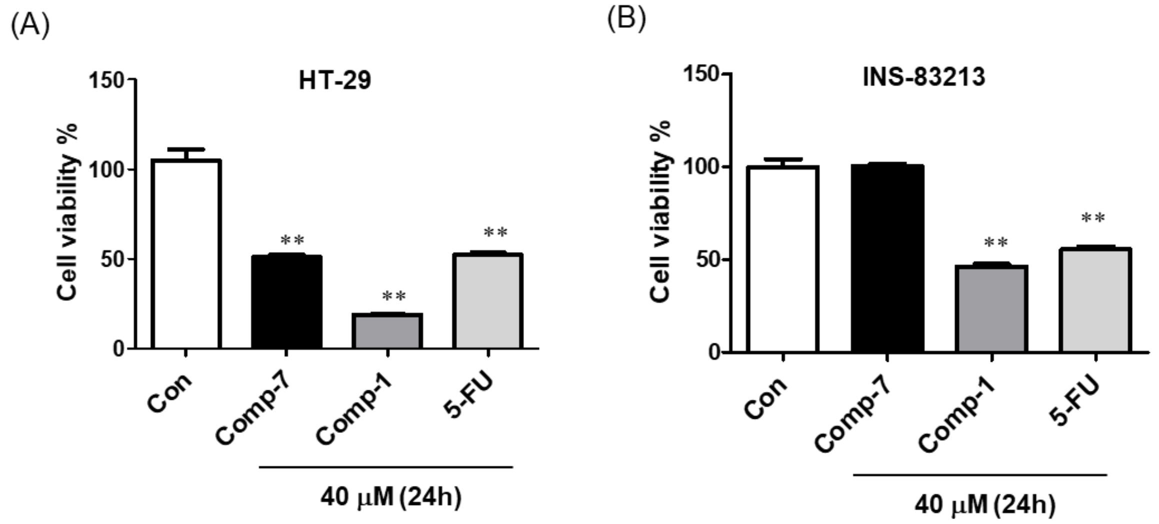

Figure S2. Cell cytotoxic effect of compound **7** in cancer and normal cell line. (A) HT-29 and (B) INS-83213 cells were plated in 96 well and treated with compound **7**, compound **1** (FTY720) and 5-fluorouracil (5-FU) 40 μM for 24 h, and cell viability was measured by EZ-CYTOTOX kit. Data are means ± SD from three independent experiments. (*n*=8) **p<0.01 compared with control cells.

^{1}H and ^{13}C NMR Spectra

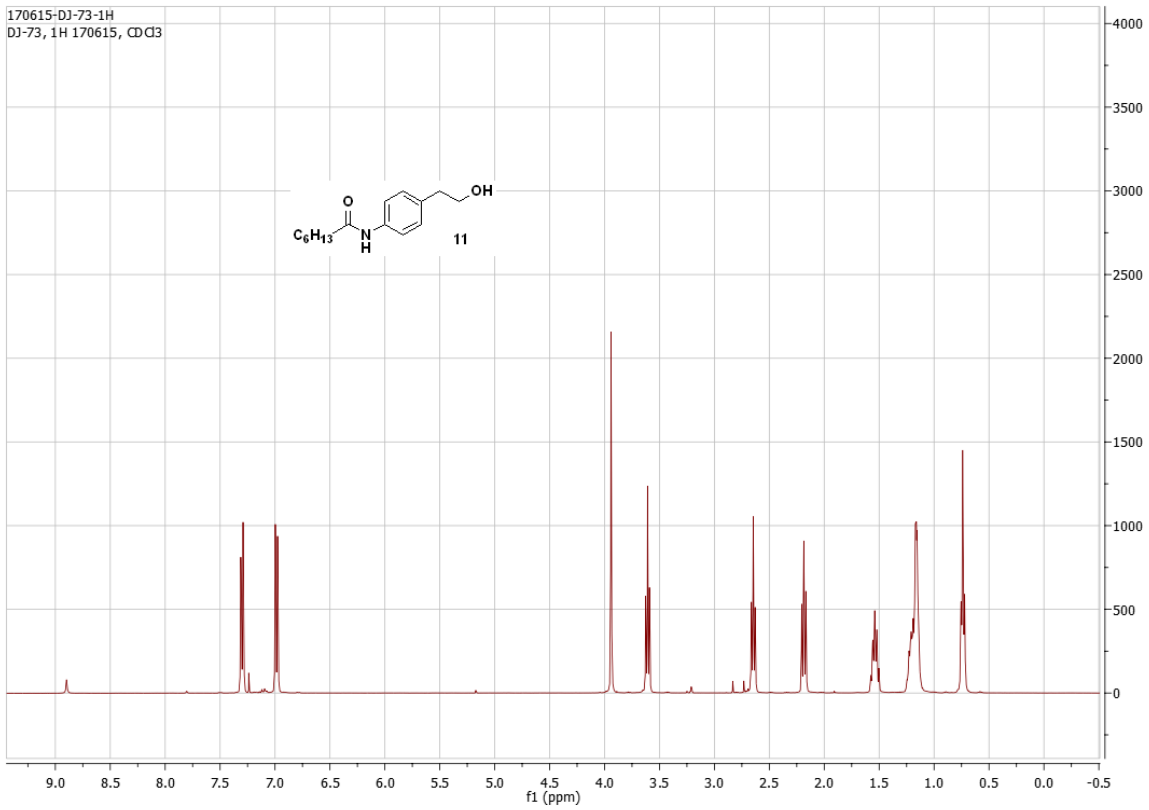

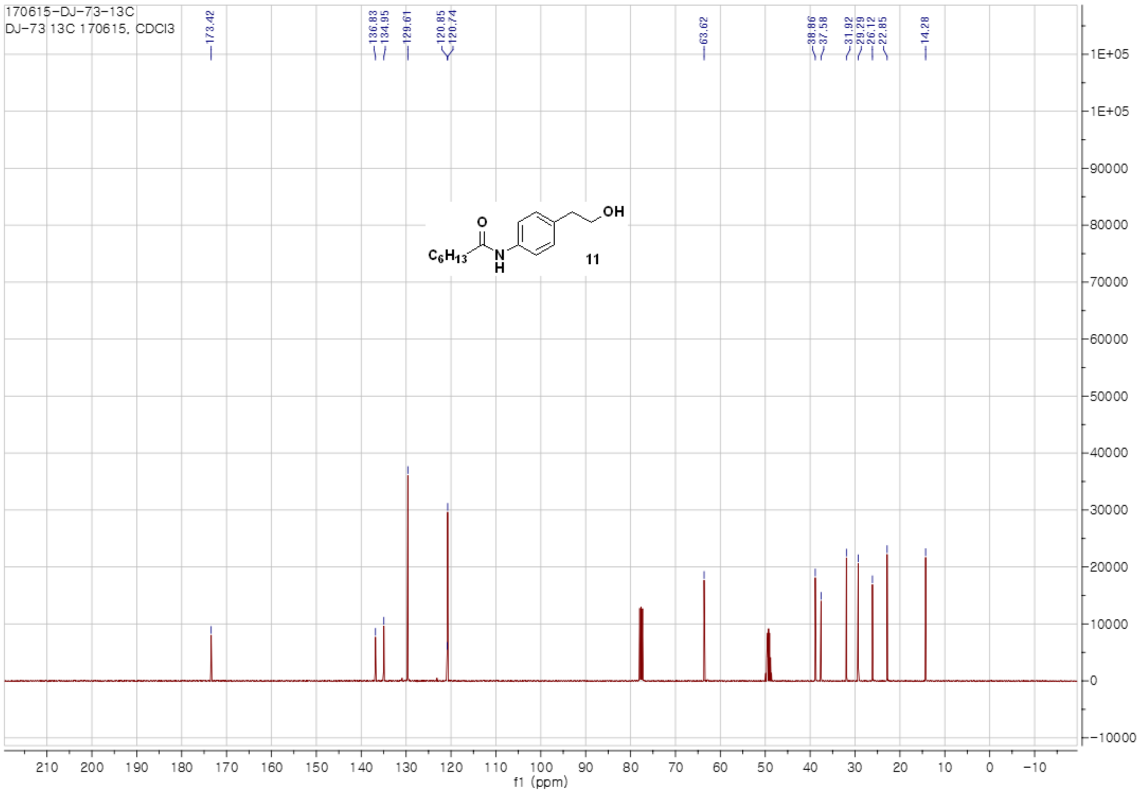

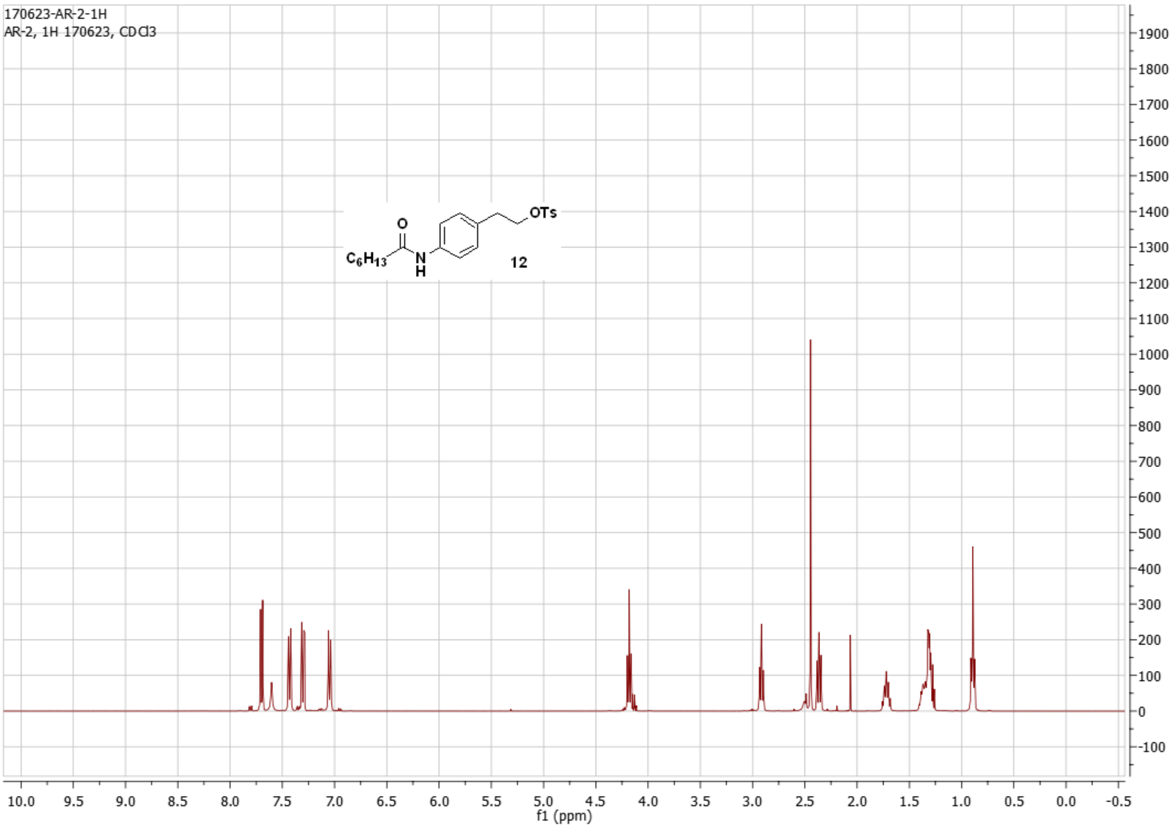
170623-AR-2-1H
AR-2, 1H 170623, CDCl3
OTs
O
C6H13
N
H
12
f1 (ppm)

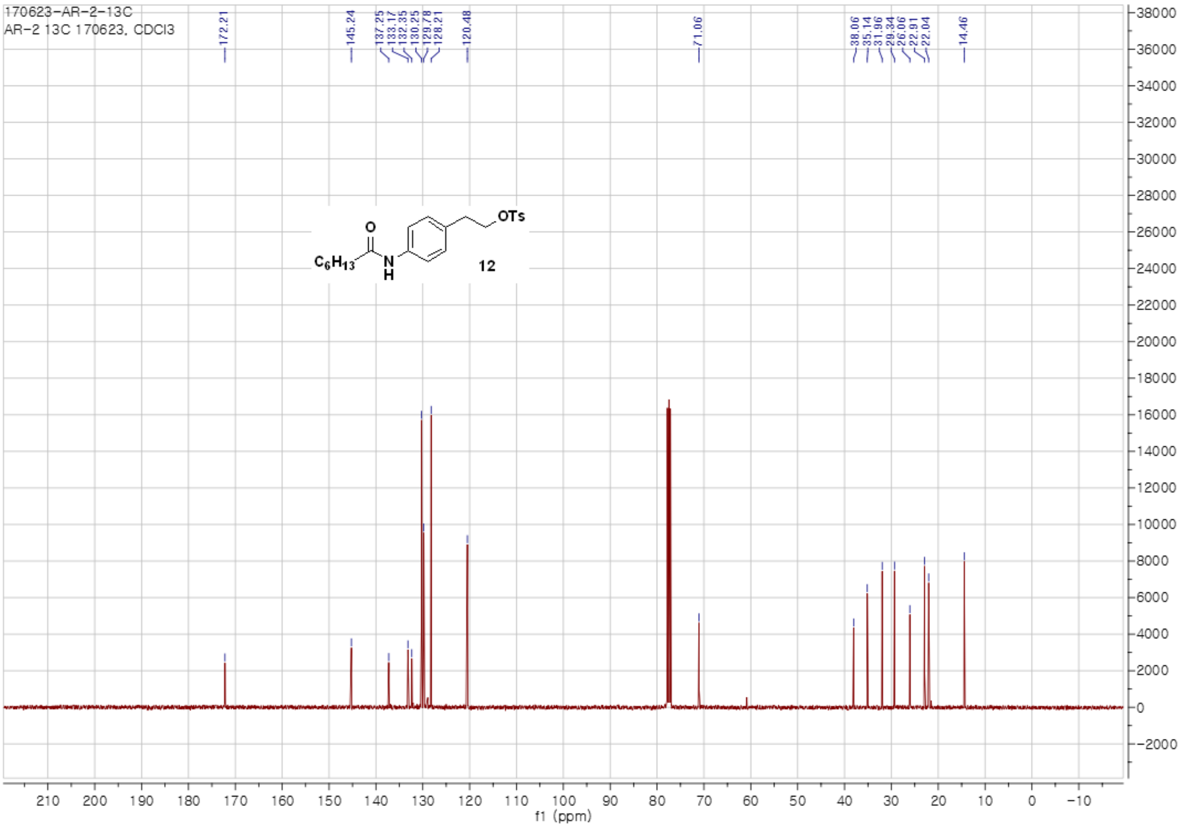
170623-AR-2-13C
AR-2 13C 170623, CDCl3
172.21
145.24
137.25
133.17
132.25
130.25
129.78
128.21
120.48
71.06
38.06
35.14
31.96
29.34
26.06
22.91
22.04
14.46
OTs
O
C6H13
N
H
12
f1 (ppm)

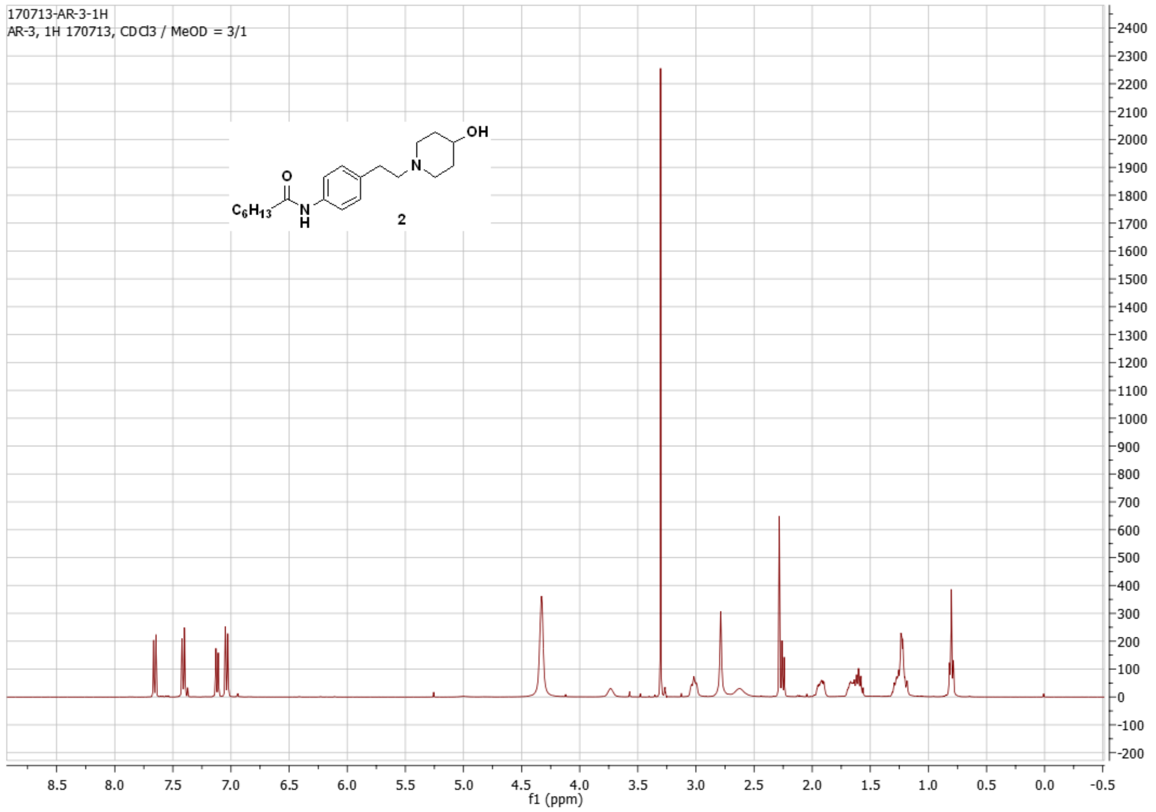
170713-AR-3-1H
AR-3, 1H 170713, CDCl3 / MeOD = 3/1
OH
N
O
C_6H_{13}
N
H
2
f1 (ppm)

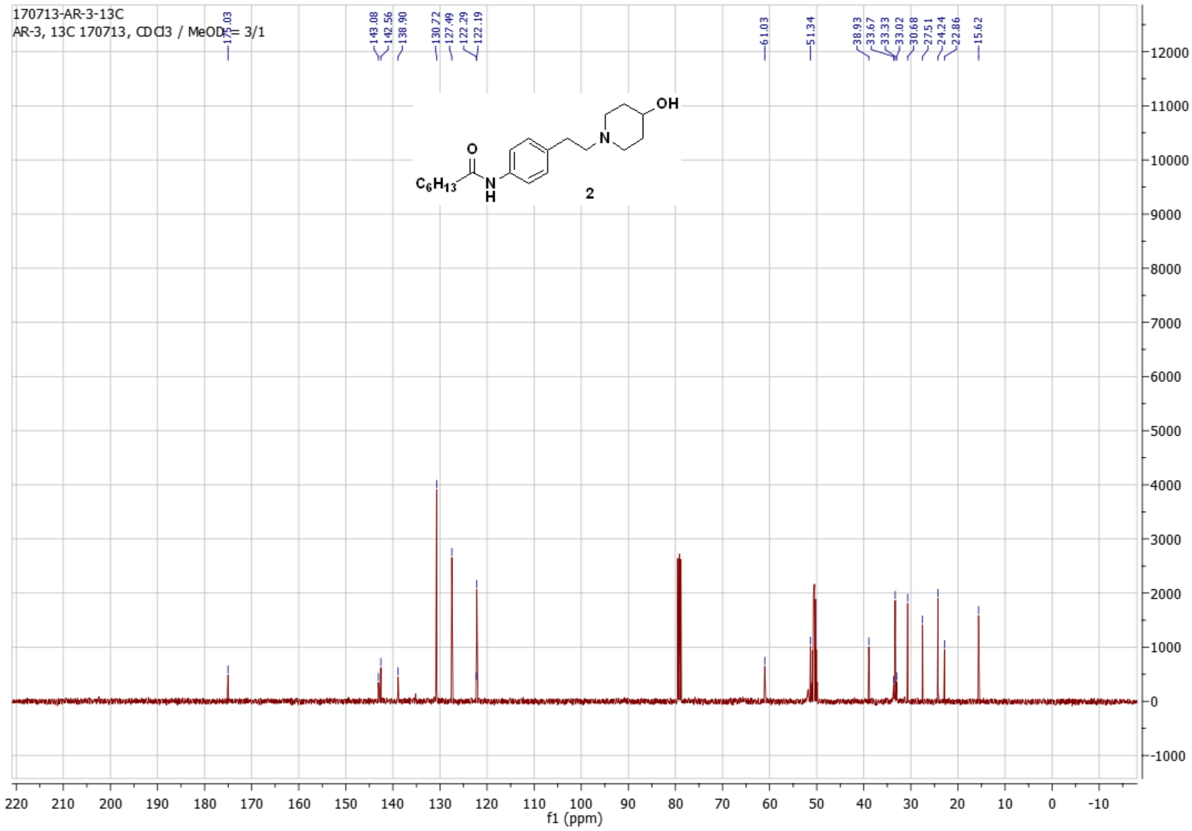
170713-AR-3-13C
AR-3, 13C 170713, CDCl3 / MeOD = 3/1
175.03
143.08
142.56
138.90
130.72
127.49
122.29
122.19
61.03
51.34
38.93
33.67
33.33
33.02
30.68
27.51
24.24
22.86
15.62
OH
N
O
C_6H_{13}
N
H
2
f1 (ppm)

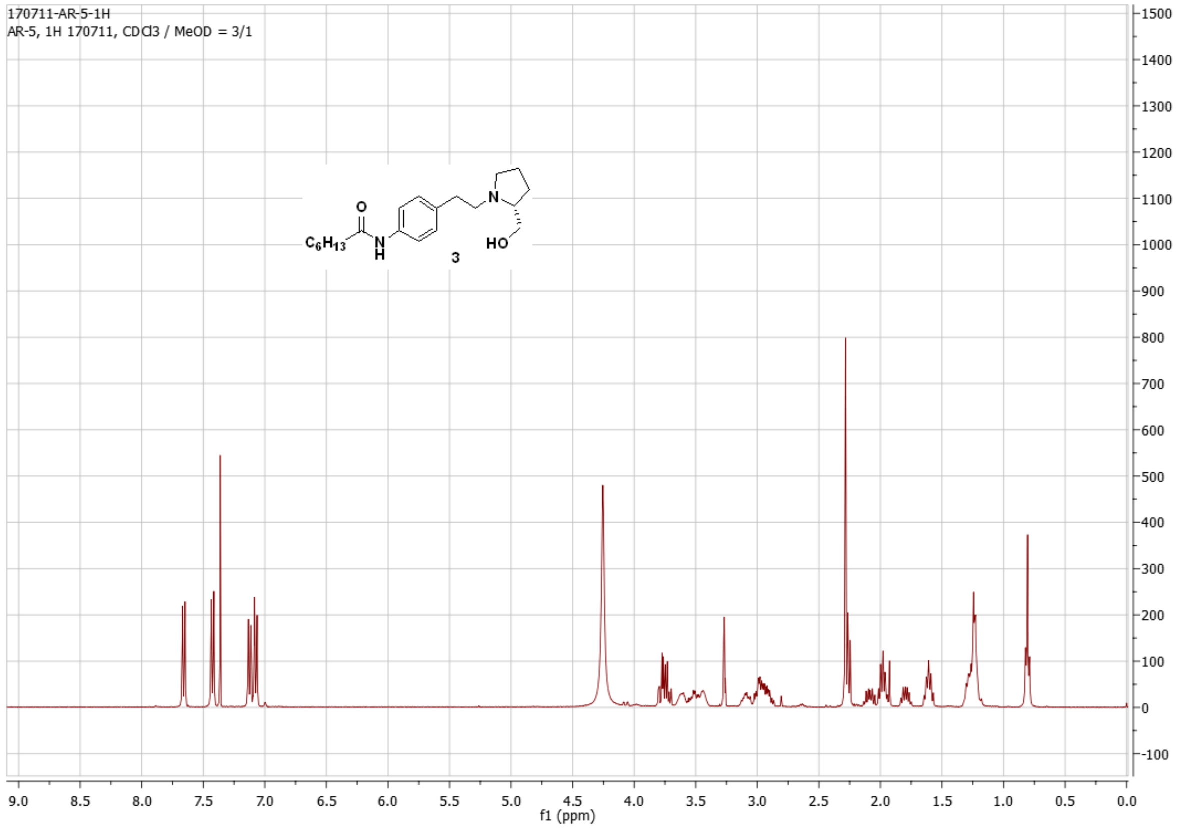

170711-AR-5-1H
AR-5, 1H 170711, CDCl3 / MeOD = 3/1
3
f1 (ppm)

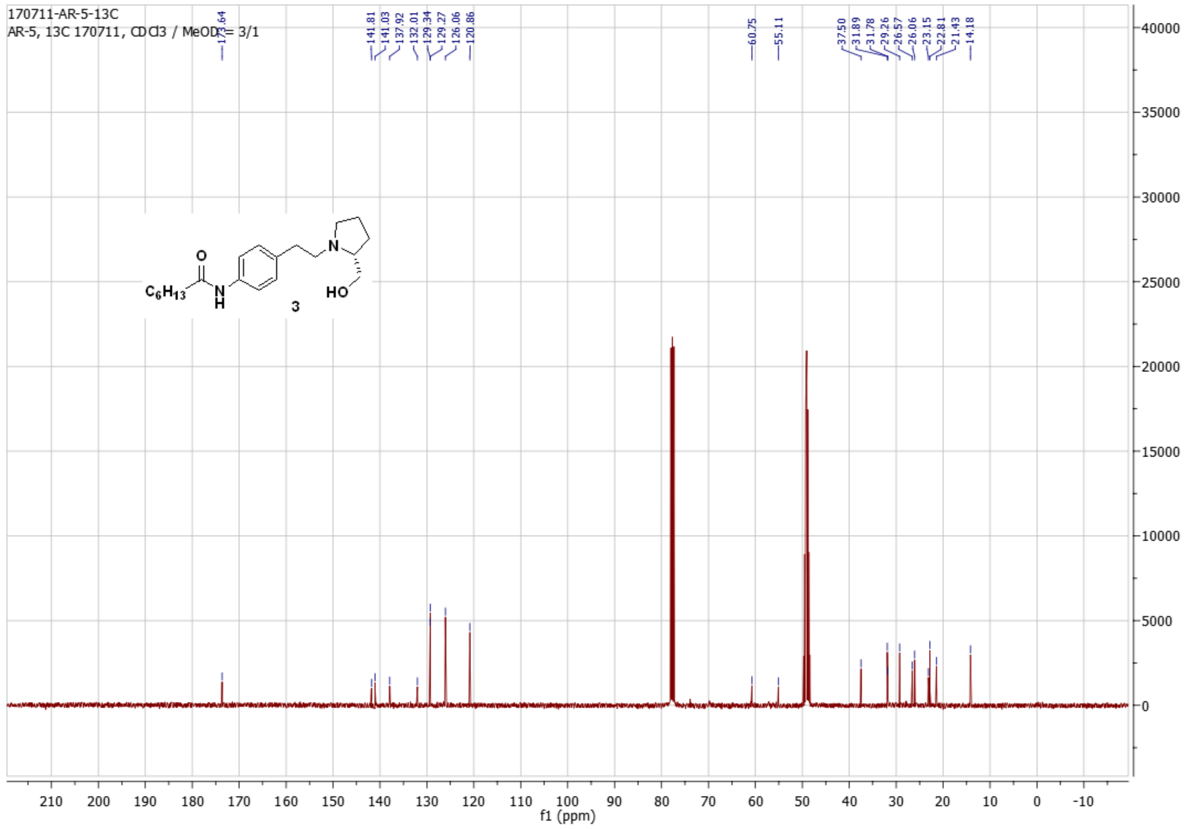

170711-AR-5-13C
AR-5, 13C 170711, CDCl3 / MeOD = 3/1
141.81
141.03
137.92
132.01
129.34
129.27
126.06
120.86
60.75
55.11
37.50
31.89
31.78
29.26
26.57
26.06
23.15
22.81
21.43
14.18
3
f1 (ppm)

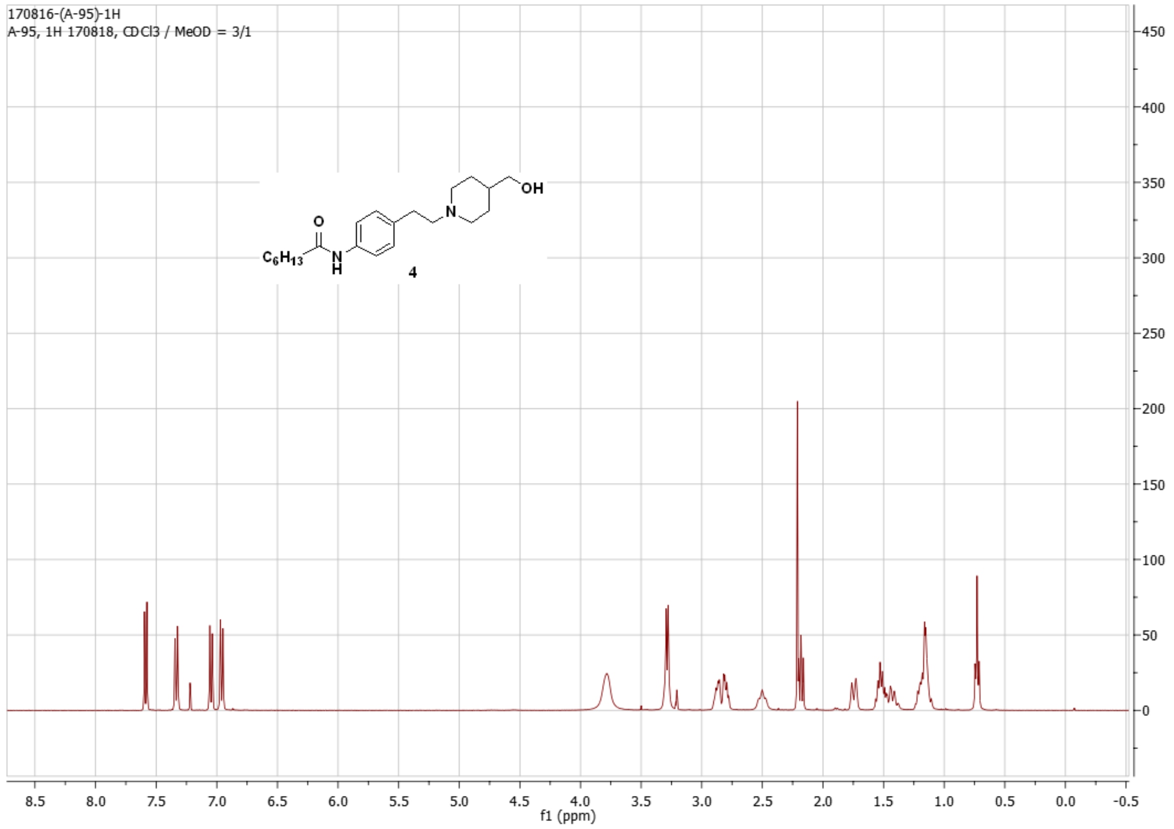
170816-(A-95)-1H
A-95, 1H 170818, CDCl3 / MeOD = 3/1
OH
N
O
C6H13
N
H
4
f1 (ppm)

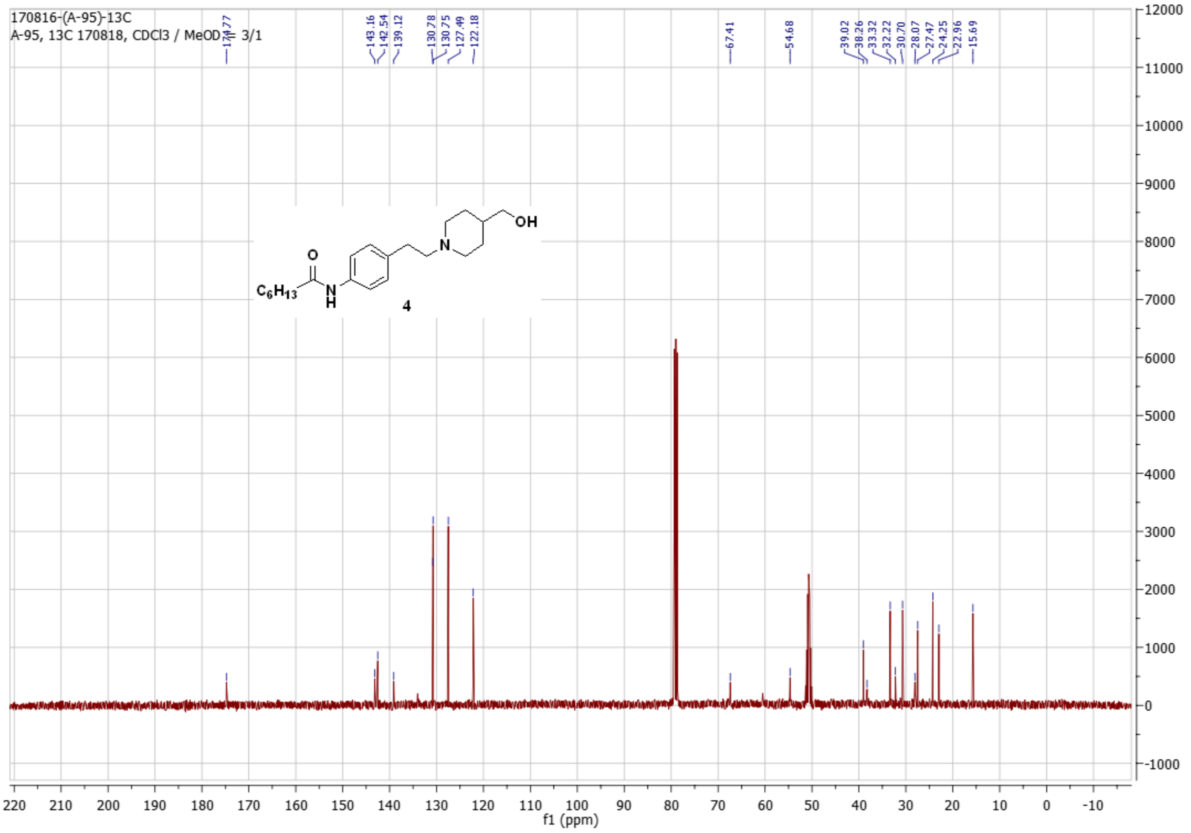
170816-(A-95)-13C
A-95, 13C 170818, CDCl3 / MeOD = 3/1
174.77
143.16
142.54
139.12
130.78
130.75
127.49
122.18
67.41
54.68
39.02
38.26
33.32
32.22
30.70
28.07
27.47
24.25
22.96
15.69
OH
N
O
C6H13
N
H
4
f1 (ppm)

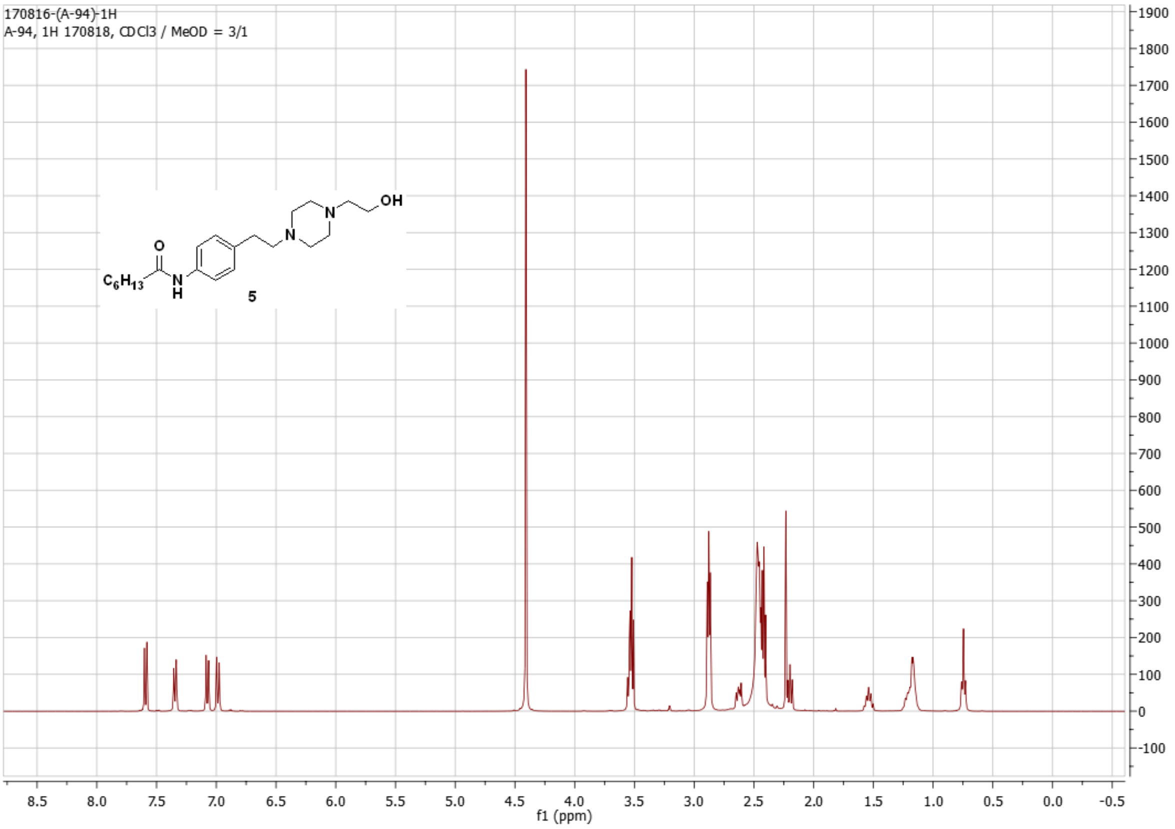
170816-(A-94)-1H
A-94, 1H 170818, CDCl3 / MeOD = 3/1
5
f1 (ppm)

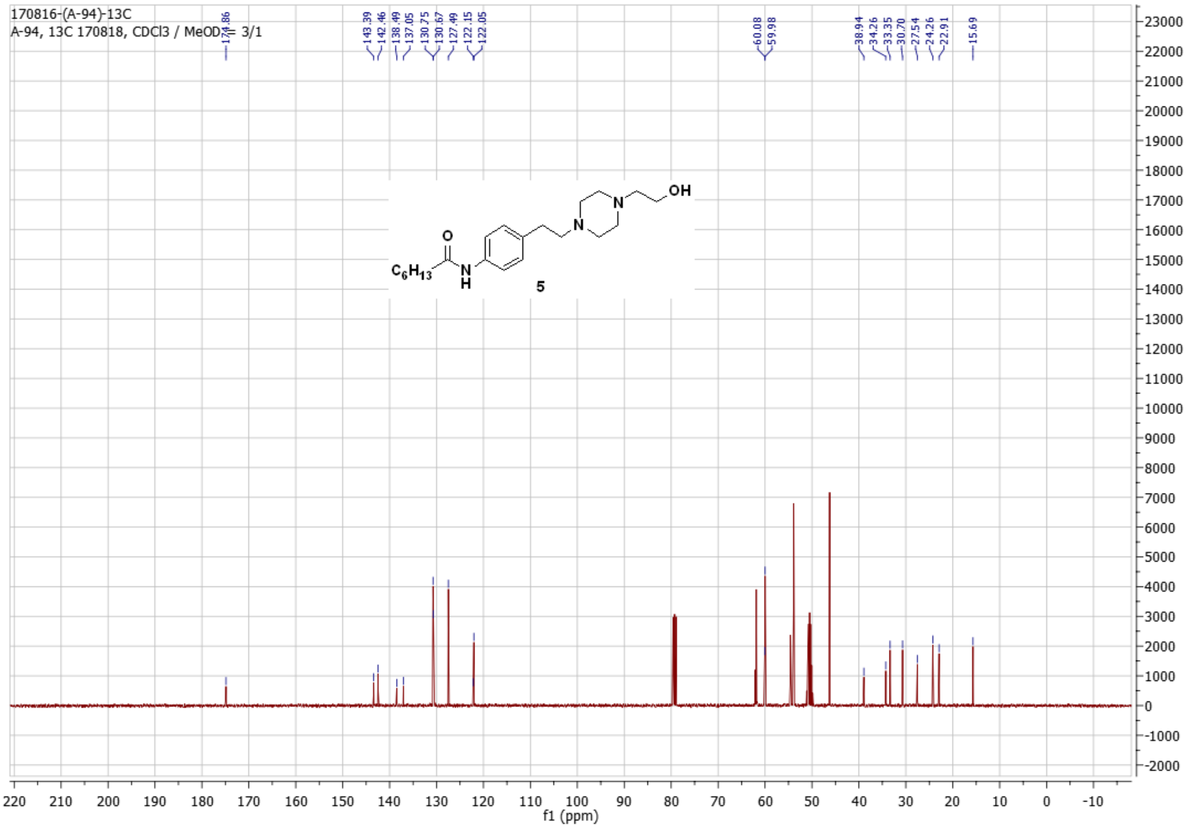
170816-(A-94)-13C
A-94, 13C 170818, CDCl3 / MeOD = 3/1
174.86
143.39
142.46
138.49
137.05
130.75
130.67
127.49
122.15
122.05
60.08
59.98
38.94
34.26
33.35
30.70
27.54
24.26
22.91
15.69
5
f1 (ppm)

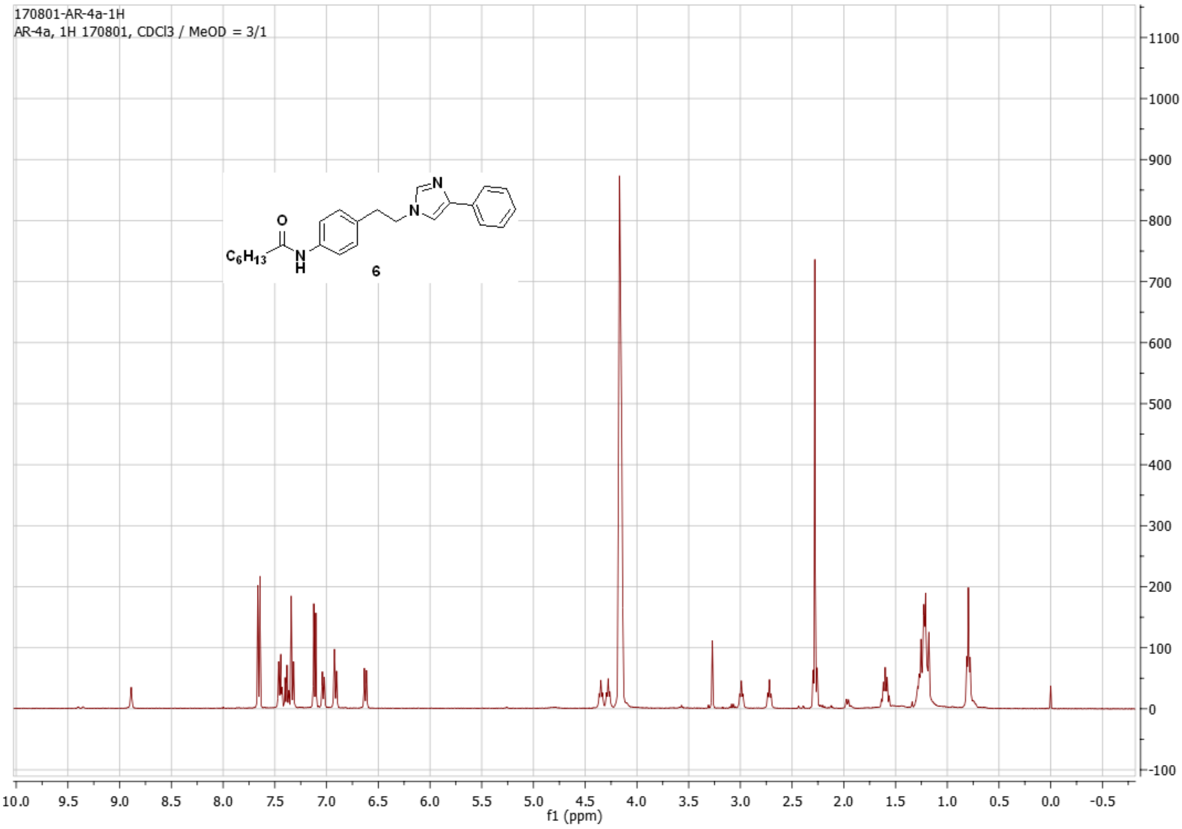

170801-AR-4a-1H
AR-4a, 1H 170801, CDCl3 / MeOD = 3/1
C_6H_{13}
O
N
H
N
N
6
f1 (ppm)

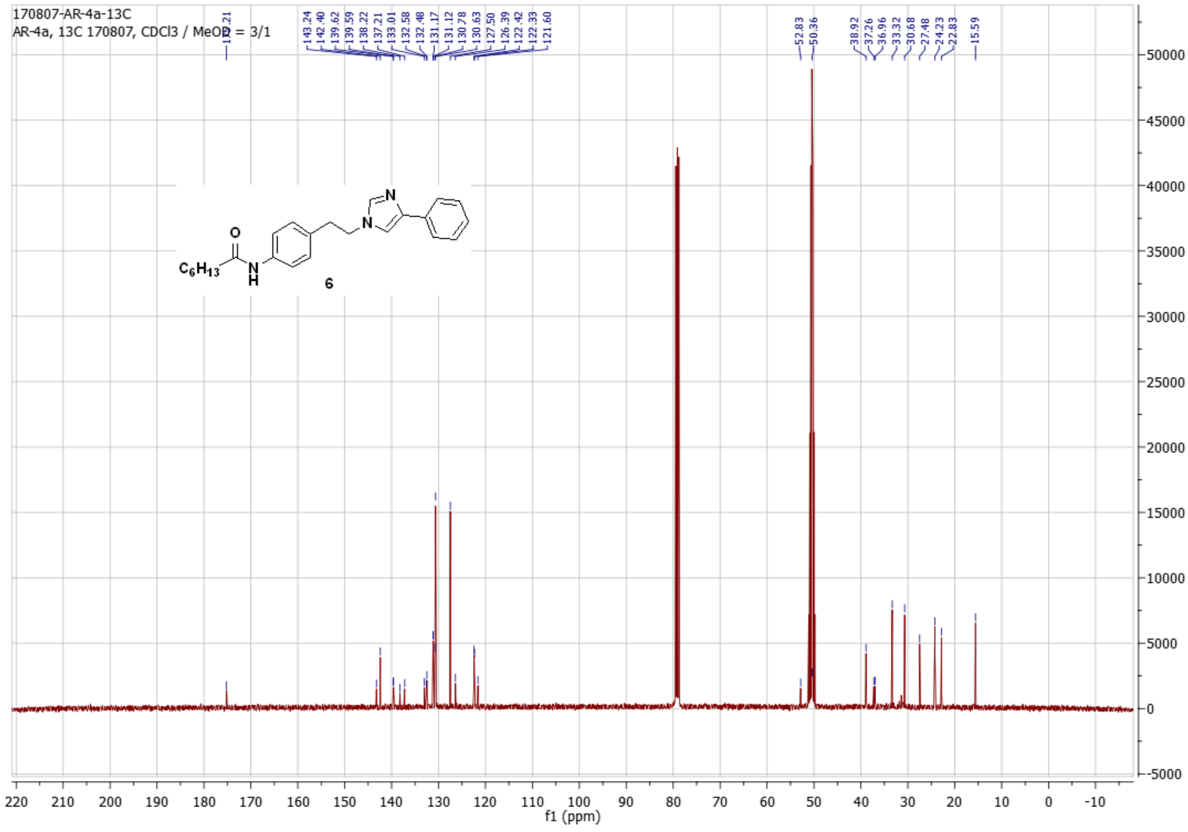

170807-AR-4a-13C
AR-4a, 13C 170807, CDCl3 / MeOD = 3/1
143.24
142.40
139.62
139.59
138.22
137.21
133.01
132.58
132.48
131.17
131.12
130.78
130.63
127.50
126.39
122.42
122.33
121.60
52.83
50.36
38.92
37.26
36.96
33.32
30.68
27.48
24.23
22.83
15.59
C_6H_{13}
O
N
H
N
N
6
f1 (ppm)

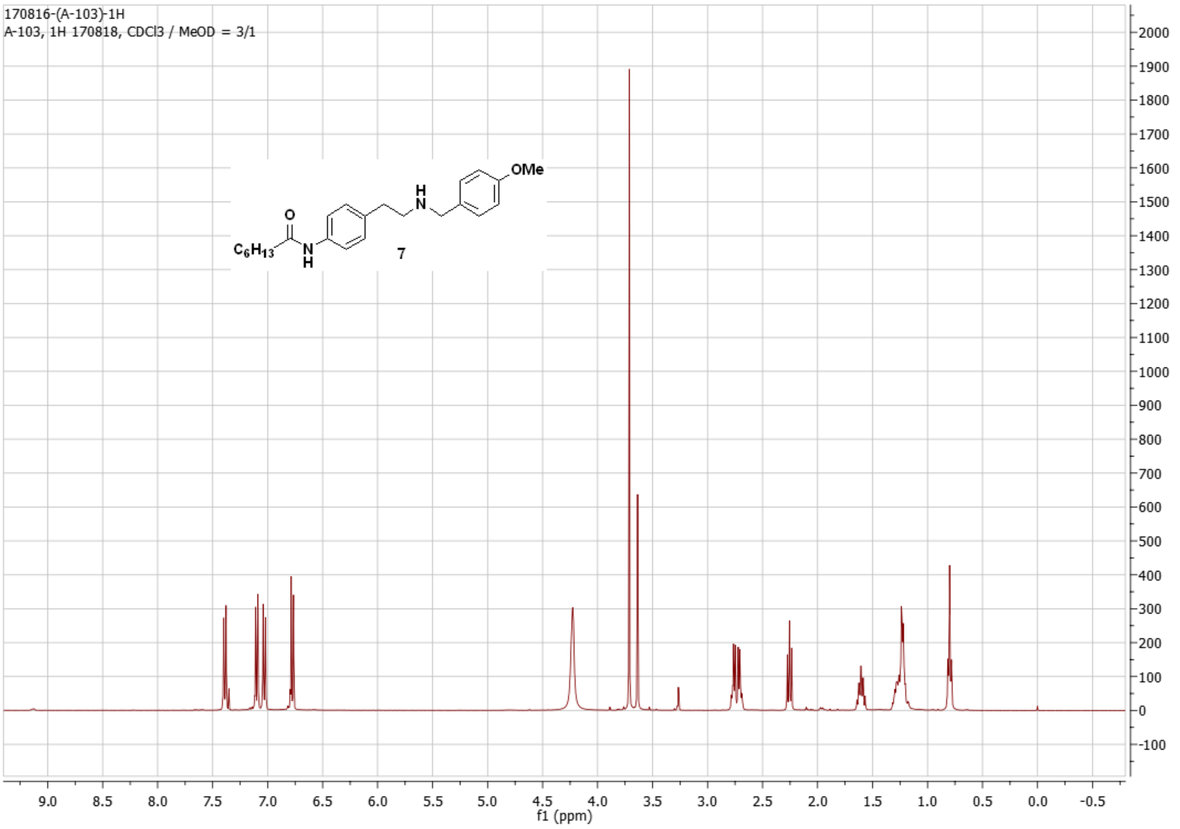
170816-(A-103)-1H
A-103, 1H 170818, CDCl3 / MeOD = 3/1
OMe
H
N
O
C_6H_{13}
N
H
7
f1 (ppm)

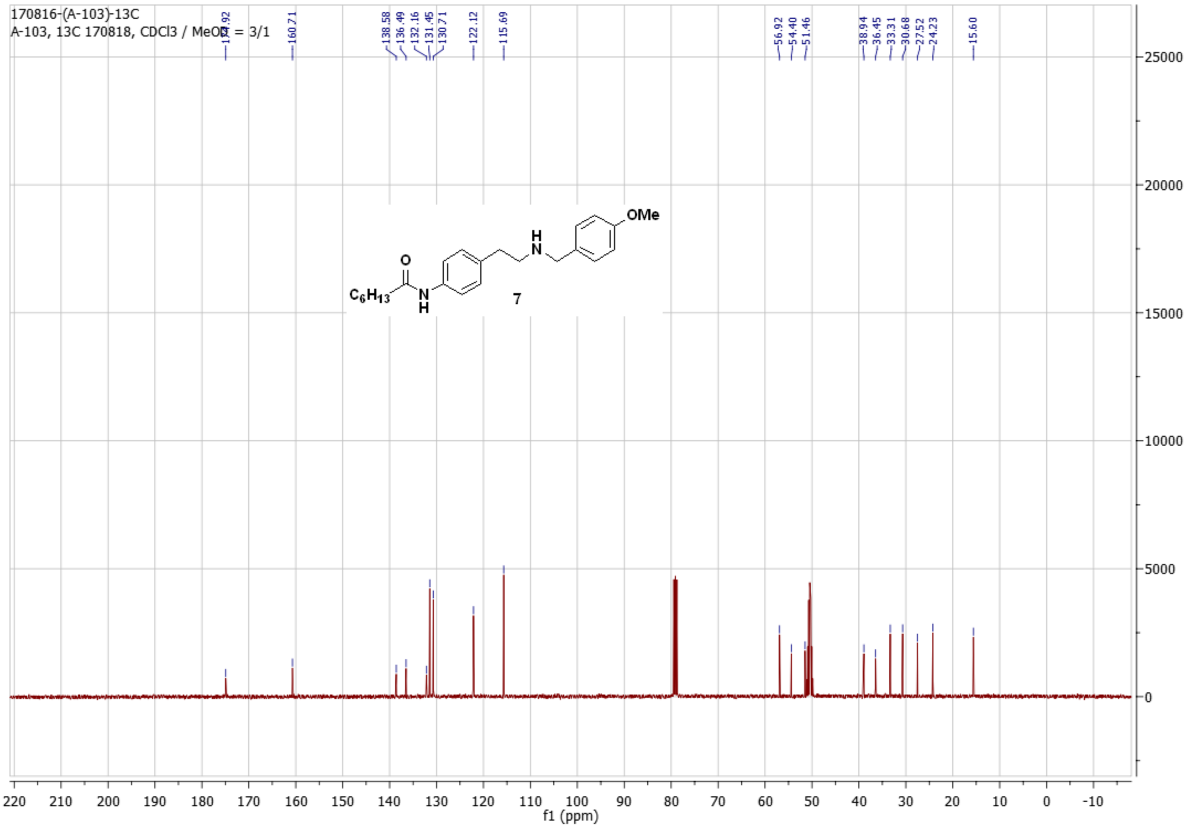
170816-(A-103)-13C
A-103, 13C 170818, CDCl3 / MeOD = 3/1
160.71
138.58
136.49
132.16
131.45
130.71
122.12
115.69
56.92
54.40
51.46
38.94
36.45
33.31
30.68
27.52
24.23
15.60
OMe
H
N
O
C_6H_{13}
N
H
7
f1 (ppm)

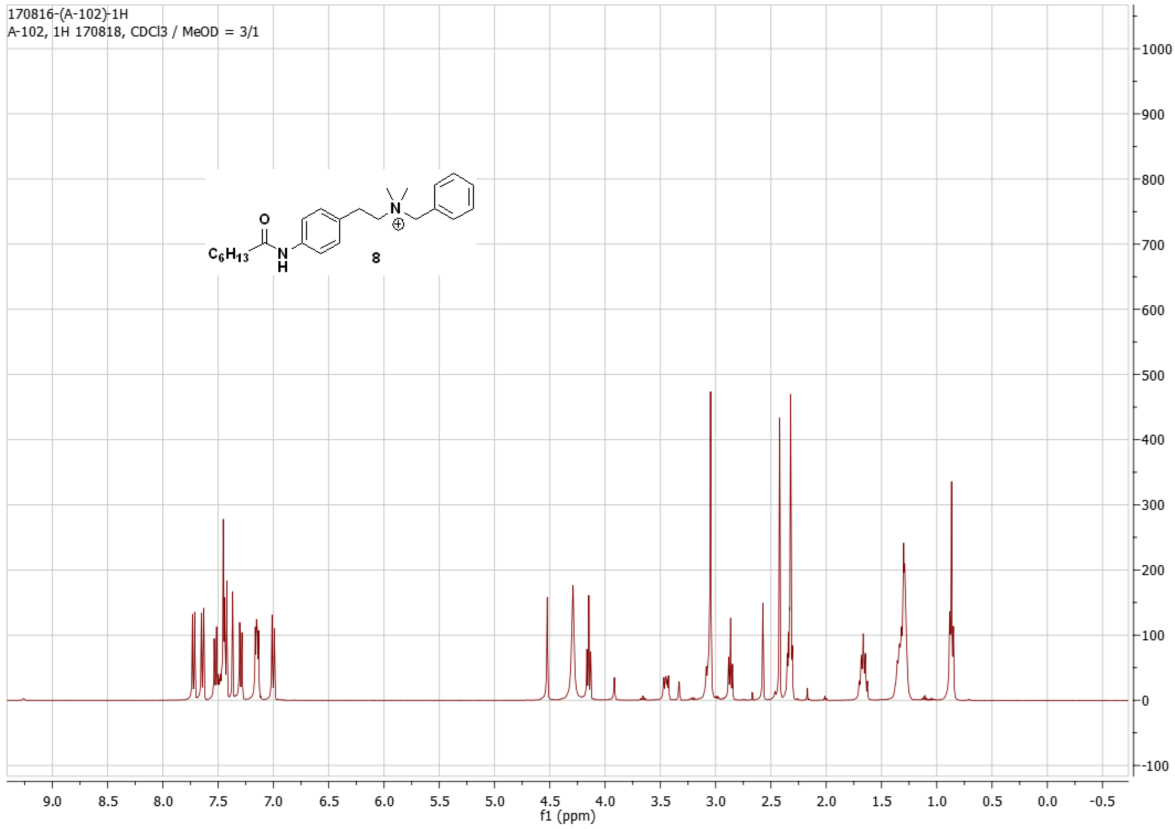

170816-(A-102)-1H
A-102, 1H 170818, CDCl3 / MeOD = 3/1
O
N
H
N
⊕
8
f1 (ppm)

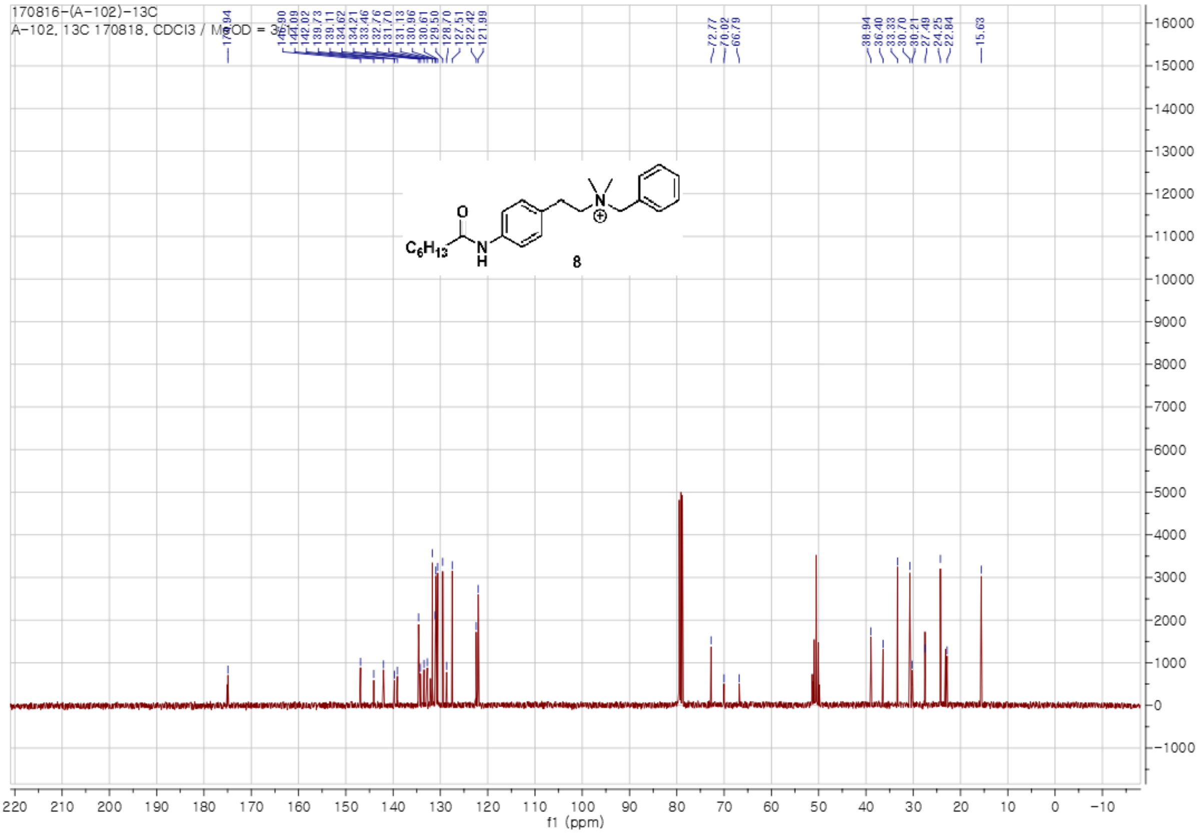

170816-(A-102)-13C
A-102, 13C 170818, CDCl3 / MeOD = 3/1
174.94
146.90
144.09
142.02
139.73
139.11
134.62
134.21
133.46
132.76
131.70
131.13
130.96
130.61
129.50
128.70
127.51
122.42
121.99
72.77
70.02
66.79
38.94
36.40
33.33
30.70
30.21
27.49
24.25
22.84
15.63
O
N
H
N
⊕
8
f1 (ppm)

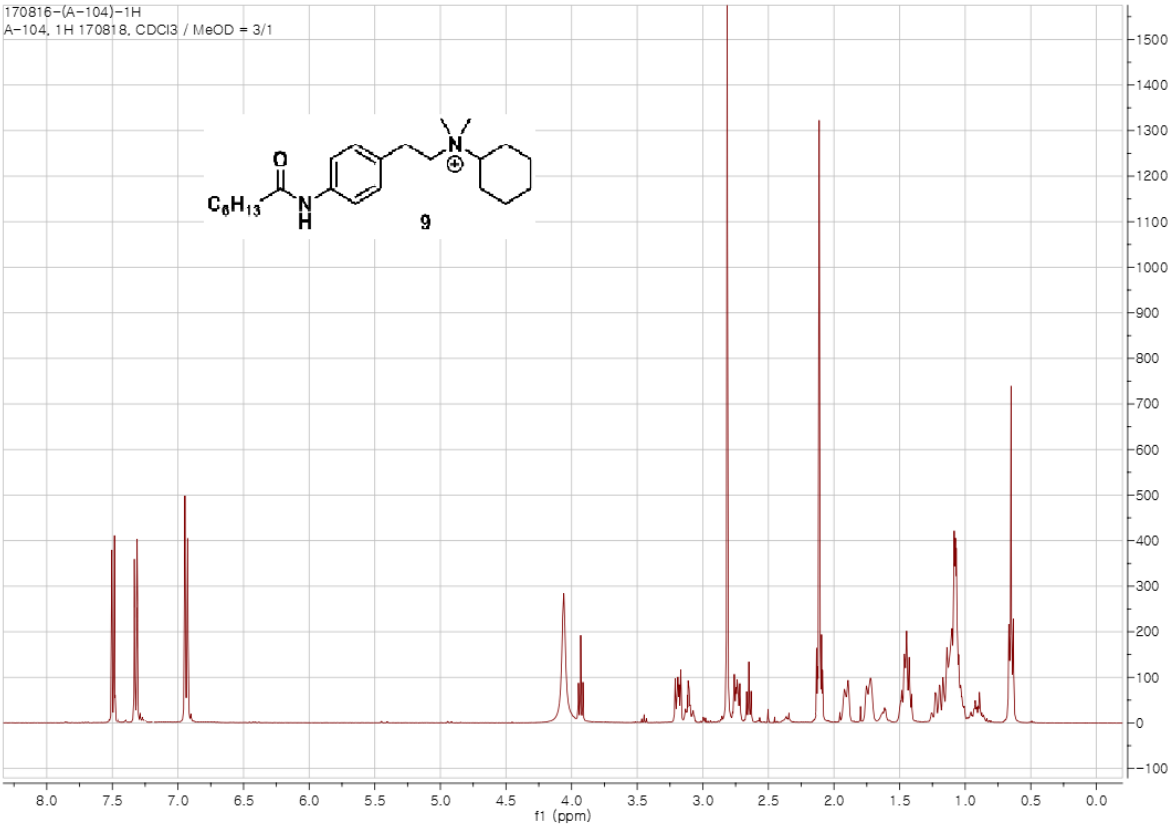
170816-(A-104)-1H
A-104, 1H 170818, CDCl3 / MeOD = 3/1
C_6H_{13}
O
N
H
N
⊕
9
f1 (ppm)

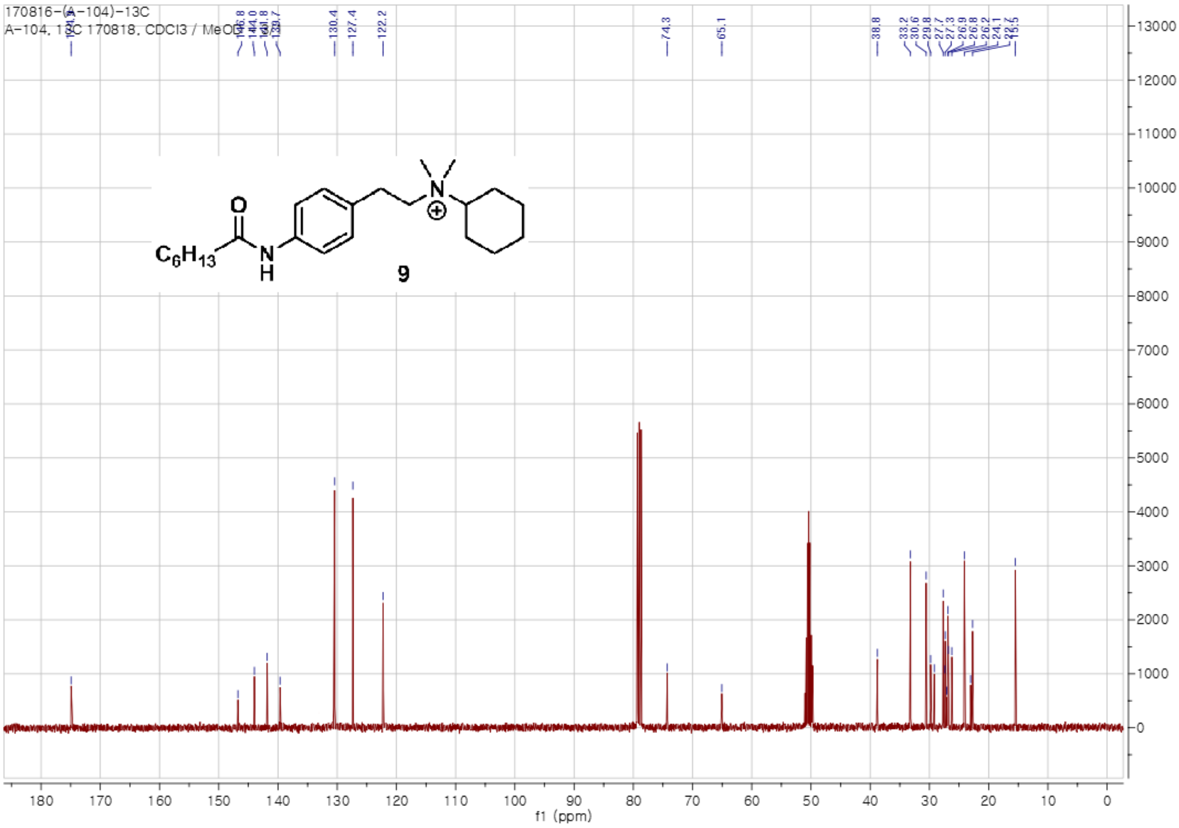
170816-(A-104)-13C
A-104, 13C 170818, CDCl3 / MeOD = 3/1
130.4
127.4
122.2
74.3
65.1
38.8
33.2
30.6
29.8
27.7
27.3
26.9
26.8
26.2
24.1
15.5
C_6H_{13}
O
N
H
N
⊕
9
f1 (ppm)

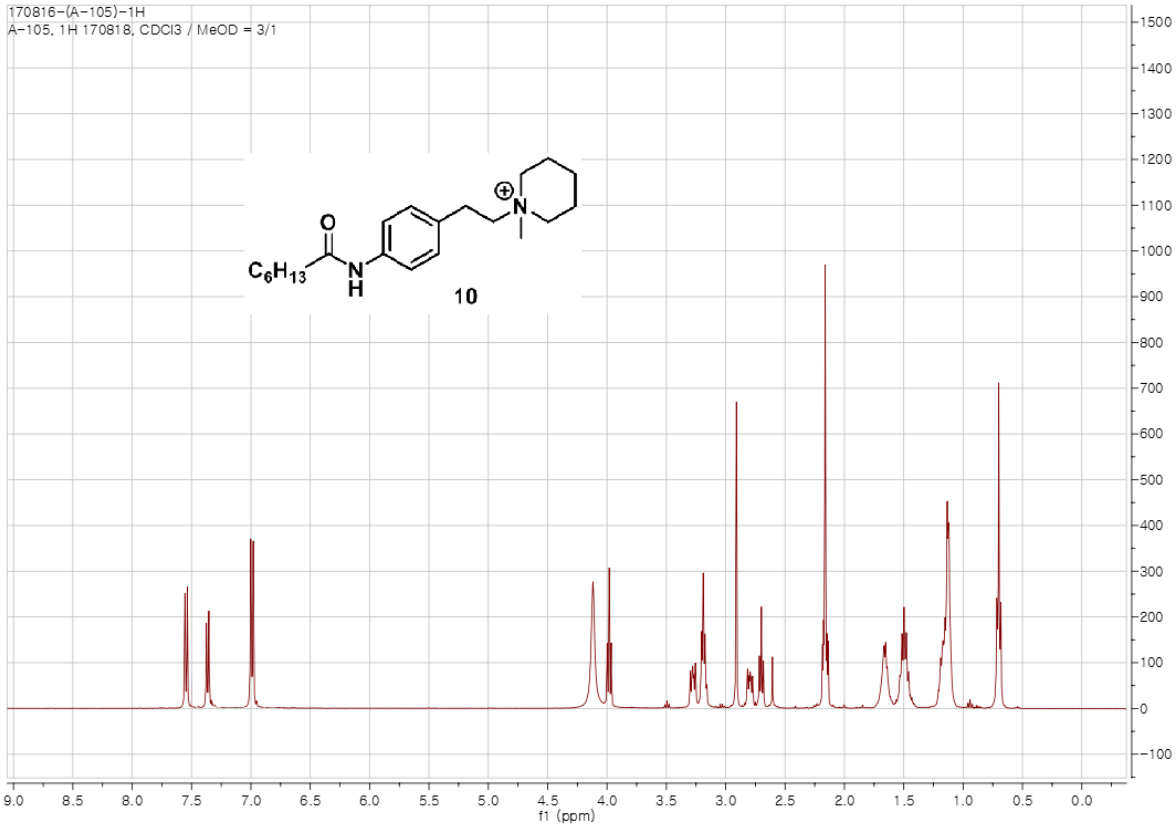
170816-(A-105)-1H
A-105, 1H 170818, CDCl3 / MeOD = 3/1
C6H13
10
f1 (ppm)

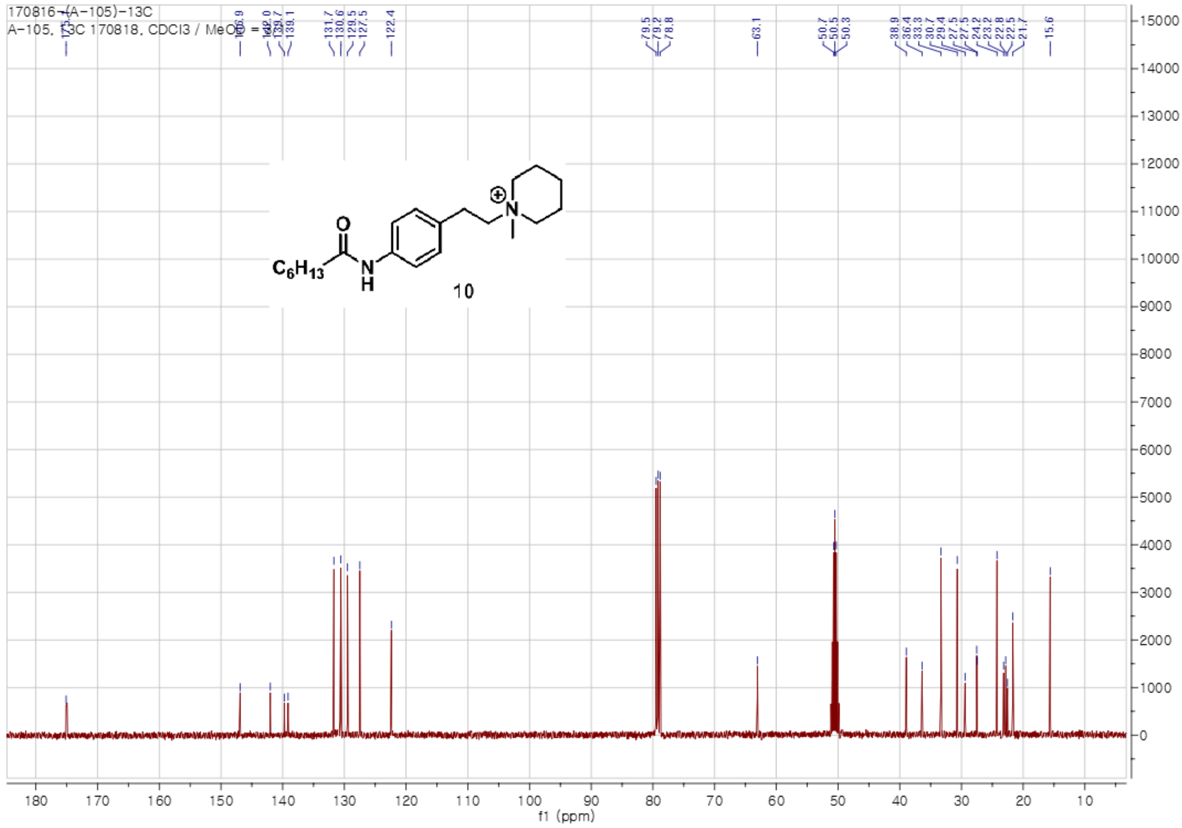
170816-(A-105)-13C
A-105, 13C 170818, CDCl3 / MeOD = 3/1
146.9
131.7
130.6
129.5
127.5
122.4
79.5
79.2
78.8
63.1
50.7
50.5
50.3
38.9
36.4
33.3
30.7
29.4
27.5
24.2
23.2
22.8
22.5
21.7
15.6
C6H13
10
f1 (ppm)

HRMS sample name: A-73 (compound 11), AR-2 (compound 12), AR-3 (compound 2), AR-5 (compound 3), A-95 (compound 4), A-94 (compound 5), AR-4 (compound 6), A-103 (compound 7), A-102 (compound 8), A-104 (compound 9), A-105 (compound 10)

**Data File**	20180410A	**Sample Name**	A-73
**Sample Type**	Sample	**Position**	
**Instrument Name**	Instrument 1	**User Name**	
**Acq Method**	HC ESI Pos Small Molecule LT No HPLC.m	**Acquired Time**	4/10/2018 4:08:50 PM
**IRM Calibration Status**	Success	**DA Method**	HCEmpirical1.m
**Comment**	EM=249.3540 M=HC ESI Pos Small Molecule LT No HPLC.m		

Sample Group **Info.**

Molecular Formula C15H23NO2

Compound Table

Compound Label	RT	Mass	Abund	Formula
Cpd 1: C15H23NO2	0.25	250.1807	42513	C15H23NO2

Compound Label	*m/z*	RT	Algorithm	Mass
Cpd 1: C15H23NO2	250.1844	0.25	Find By Formula	250.1807

MS Spectrum

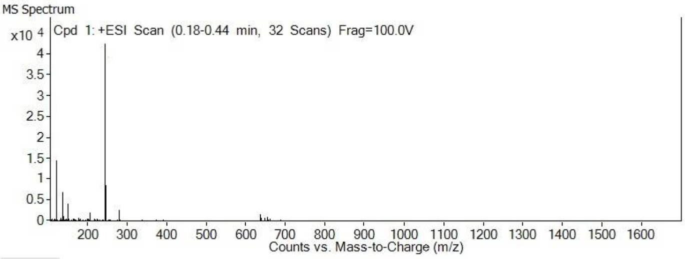

**Data File**	20180418F	**Sample Name**	AR-2
**Sample Type**	Sample	**Position**	
**Instrument Name**	Instrument 1	**User Name**	
**Acq Method**	No HPLC MeOH 1.m	**Acquired Time**	4/18/2018 5:31:01 PM
**IRM Calibration Status**	Success	**DA Method**	Data 1.m
**Comment**	EM=403.5370 M=No HPLC MeOH 1.m		

Sample Group **Info.**

Molecular Formula C22H29NO4S **Acquisition SW Version** 6200 series TOF/6500 series Q-TOF B.05.01 (B5125.1)

Compound Table

Compound Label	RT	Mass	Abund	Formula
Cpd 1: C22H29NO4S	0.182	404.1896	192637	C22H29NO4S

Compound Label	*m/z*	RT	Algorithm	Mass
Cpd 1: C22H29NO4S	404.1858	0.182	Find By Formula	404.1896

MS Spectrum

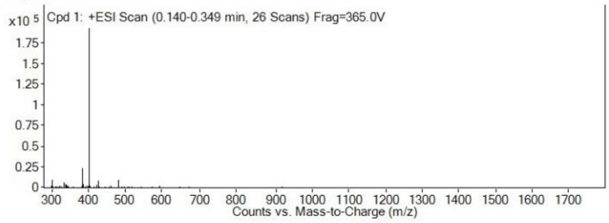

**Data File**	20180501A	**Sample Name**	AR-3
**Sample Type**	Sample	**Position**	
**Instrument Name**	Instrument 1	**User Name**	
**Acq Method**	HC ESI Pos Small Molecule LT No HPLC.m	**Acquired Time**	5/1/2018 9:39:49 AM
**IRM Calibration Status**	Success	**DA Method**	HCEmpirical1.m
**Comment**	EM=332.4880 M=HC ESI Pos Small Molecule LT No HPLC.m		

Sample Group **Info.**

Molecular Formula C20H32N2O2

Compound Table

Compound Label	RT	Mass	Abund	Formula
Cpd 1: C20H32N2O2	0.23	333.2542	21580	C20H32N2O2

Compound Label	*m/z*	RT	Algorithm	Mass
Cpd 1: C20H32N2O2	333.2575	0.23	Find By Formula	333.2542

MS Spectrum

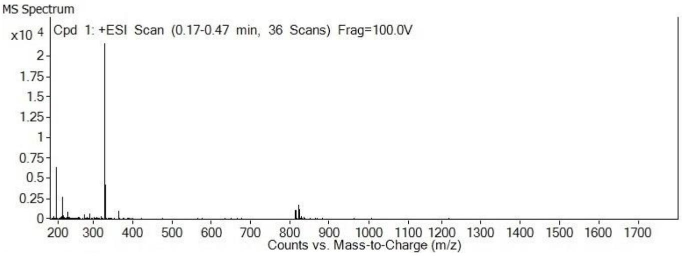

**Data File**	20180501C	**Sample Name**	AR-5
**Sample Type**	Sample	**Position**	
**Instrument Name**	Instrument 1	**User Name**	
**Acq Method**	No HPLC MeOH 1.m	**Acquired Time**	5/1/2018 10:22:01 AM
**IRM Calibration Status**	Success	**DA Method**	Data 1.m
**Comment**	EM=332.4880 M=No HPLC MeOH 1.m		

**Sample Group**		**Info.**	
**Molecular Formula**	C20H32N2O2	**Acquisition SW Version**	6200 series TOF/6500 series Q-TOF B.05.01 (B5125)

Compound Table

Compound Label	RT	Mass	Abund	Formula
Cpd 1: C20H32N2O2	0.186	333.2542	138368	C20H32N2O2

Compound Label	*m/z*	RT	Algorithm	Mass
Cpd 1: C20H32N2O2	333.2517	0.186	Find By Formula	333.2542

MS Spectrum

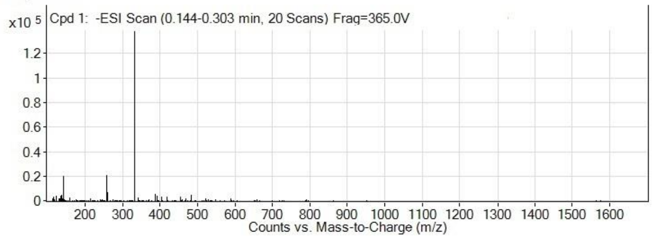

**Data File**	20180501B	**Sample Name**	AR-4
**Sample Type**	Sample	**Position**	
**Instrument Name**	Instrument 1	**User Name**	
**Acq Method**	No HPLC MeOH 1.m	**Acquired Time**	5/1/2018 9:52:55 AM
**IRM Calibration Status**	Success	**DA Method**	Data 1.m
**Comment**	EM=475.5160 M=No HPLC MeOH 1.m		

**Sample Group**		**Info.**	
**Molecular Formula**	C24H29N3O	**Acquisition SW Version**	6200 series TOF/6500 series Q-TOF B.05.01 (B5125)

Compound Table

Compound Label	RT	Mass	Abund	Formula
Cpd 1: C24H29N3O	0.179	376.2389	614304	C24H29N3O

Compound Label	*m/z*	RT	Algorithm	Mass
Cpd 1: C24H29N3O	376.2391	0.179	Find By Formula	376.2389

MS Spectrum

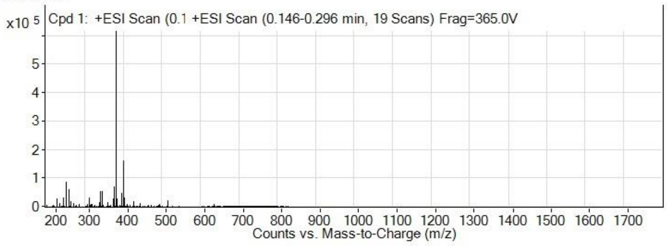

**Data File**	20180509A	**Sample Name**	A-94
**Sample Type**	Sample	**Position**	
**Instrument Name**	Instrument 1	**User Name**	
**Acq Method**	No HPLC MeOH 1.m	**Acquired Time**	5/9/2018 9:17:02 AM
**IRM Calibration Status**	Success	**DA Method**	Data 1.m
**Comment**	EM=361.5300 M=No HPLC MeOH 1.m		

**Sample Group**		**Info.**	
**Molecular Formula**	C21H35N3O2	**Acquisition SW Version**	6200 series TOF/6500 series Q-TOF B.05.01 (B5125.1)

Compound Table

Compound Label	RT	Mass	Abund	Formula
Cpd 1: C21H35N3O2	0.185	362.2808	276800	C21H35N3O2

Compound Label	*m/z*	RT	Algorithm	Mass
Cpd 1: C21H35N3O2	362.2833	0.185	Find By Formula	362.2808

MS Spectrum

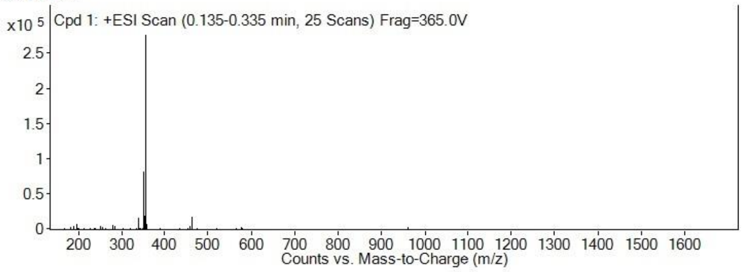

**Data File**	20180509B	**Sample Name**	A-95
**Sample Type**	Sample	**Position**	
**Instrument Name**	Instrument 1	**User Name**	
**Acq Method**	No HPLC MeOH 1.m	**Acquired Time**	5/9/2019 9:44:12 AM
**IRM Calibration Status**	Success	**DA Method**	Data 1.m
**Comment**	EM=346.5150 M=No HPLC MeOH 1.m		

**Sample Group**		**Info.**	
**Molecular Formula**	C21H34N2O2	**Acquisition SW Version**	6200 series TOF/6500 series Q-TOF B.05.01 (B5125.1)

Compound Table

Compound Label	RT	Mass	Abund	Formula
Cpd 1: C21H34N2O2	0.181	347.2699	297920	C21H34N2O2

Compound Label	*m/z*	RT	Algorithm	Mass
Cpd 1: C21H34N2O2	347.2629	0.181	Find By Formula	347.2699

MS Spectrum

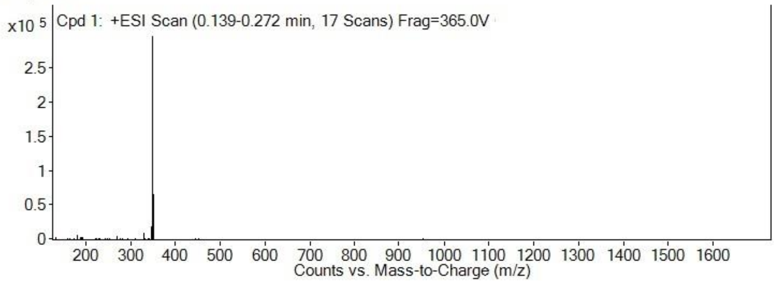

**Data File**	20180509C	**Sample Name**	A-102
**Sample Type**	Sample	**Position**	
**Instrument Name**	Instrument 1	**User Name**	
**Acq Method**	No HPLC MeOH 1.m	**Acquired Time**	5/9/2018 10:11:49 AM
**IRM Calibration Status**	Success	**DA Method**	Data 1.m
**Comment**	EM=367.5565 M=No HPLC MeOH 1.m		

**Sample Group**		**Info.**	
**Molecular Formula**	C24H35N2O	**Acquisition SW Version**	6200 series TOF/6500 series Q-TOF B.05.01 (B5125.1)

Compound Table

Compound Label	RT	Mass	Abund	Formula
Cpd 1: C24H35N2O	0.181	368.2822	391837	C24H35N2O

Compound Label	*m/z*	RT	Algorithm	Mass
Cpd 1: C24H35N2O	368.2809	0.181	Find By Formula	368.2822

MS Spectrum

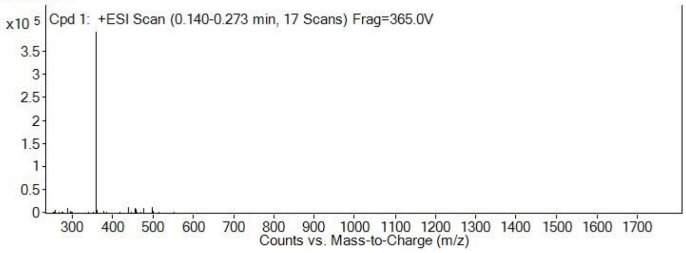

**Data File**	20180509D	**Sample Name**	A-103
**Sample Type**	Sample	**Position**	
**Instrument Name**	Instrument 1	**User Name**	
**Acq Method**	No HPLC MeOH 1.m	**Acquired Time**	5/9/2018 10:38:25 AM
**IRM Calibration Status**	Success	**DA Method**	Data 1.m
**Comment**	EM=368.5210 M=No HPLC MeOH 1.m		

Sample Group

Molecular Formula C23H32N2O2

Info.

Acquisition SW Version 6200 series TOF/6500 series Q-TOF B.05.01 (B5125.1)

Compound Table

Compound Label	RT	Mass	Abund	Formula
Cpd 1: C23H32N2O2	0.18	369.2542	249707	C23H32N2O2

Compound Label	*m/z*	RT	Algorithm	Mass
Cpd 1: C23H32N2O2	369.2538	0.18	Find By Formula	369.2542

MS Spectrum

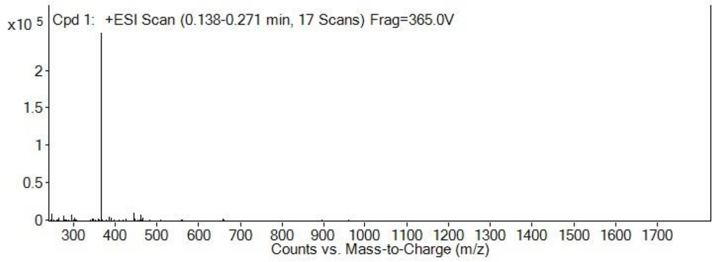

**Data File**	20180509E	**Sample Name**	A-104
**Sample Type**	Sample	**Position**	
**Instrument Name**	Instrument 1	**User Name**	
**Acq Method**	No HPLC MeOH 1.m	**Acquired Time**	5/9/2018 11:01:15 AM
**IRM Calibration Status**	Success	**DA Method**	Data 1.m
**Comment**	EM=359.5775 M=No HPLC MeOH 1.m		

Sample Group

Molecular Formula C23H39N2O

Info.

Acquisition SW Version 6200 series TOF/6500 series Q-TOF B.05.01 (B5125.1)

Compound Table

Compound Label	RT	Mass	Abund	Formula
Cpd 1: C23H39N2O	0.181	360.3135	337012	C23H39N2O

Compound Label	*m/z*	RT	Algorithm	Mass
Cpd 1: C23H39N2O	360.3123	0.181	Find By Formula	360.3135

MS Spectrum

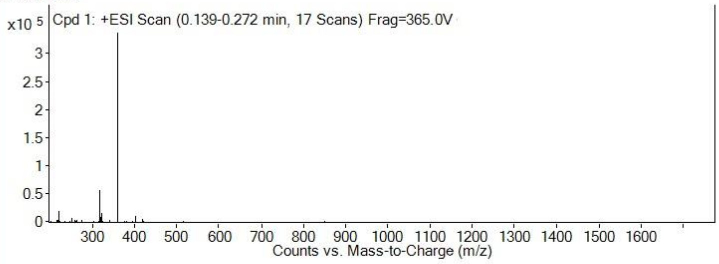

**Data File**	20180509F	**Sample Name**	A105
**Sample Type**	Sample	**Position**	
**Instrument Name**	Instrument 1	**User Name**	
**Acq Method**	No HPLC MeOH 1.m	**Acquired Time**	5/9/2018 11:27:40 AM
**IRM Calibration Status**	Success	**DA Method**	Data 1.m
**Comment**	EM=331.5235 M=No HPLC MeOH 1.m		

**Sample Group**		**Info.**	
**Molecular Formula**	C21H35N2O	**Acquisition SW Version**	6200 series TOF/6500 series Q-TOF B.05.01 (B5125.1)

Compound Table

Compound Label	RT	Mass	Abund	Formula
Cpd 1: C21H35N2O	0.176	332.2822	260882	C21H35N2O

Compound Label	*m/z*	RT	Algorithm	Mass
Cpd 1: C21H35N2O	332.2871	0.171	Find By Formula	332.2822

MS Spectrum

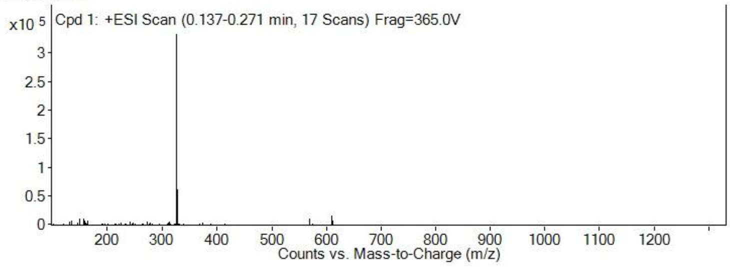

Supplement: Supplementary file 1 [file molecules-23-02750-s001.pdf]
